# Mitogen-Activated Protein Kinase (MAPK) Pathway Regulates Branching by Remodeling Epithelial Cell Adhesion

**DOI:** 10.1371/journal.pgen.1004193

**Published:** 2014-03-06

**Authors:** Anneliis Ihermann-Hella, Maria Lume, Ilkka J. Miinalainen, Anniina Pirttiniemi, Yujuan Gui, Johan Peränen, Jean Charron, Mart Saarma, Frank Costantini, Satu Kuure

**Affiliations:** 1 Institute of Biotechnology, University of Helsinki, Helsinki, Finland; 2 Department of Pathology, University of Oulu, Oulu, Finland; 3 Centre de Recherche en Cancérologie de l'Université Laval, CRCHUQ, Hôtel-Dieu de Québec, Québec, Canada; 4 Department of Genetics and Development, Columbia University Medical Center, New York, New York, United States of America; University of California San Francisco, United States of America

## Abstract

Although the growth factor (GF) signaling guiding renal branching is well characterized, the intracellular cascades mediating GF functions are poorly understood. We studied mitogen-activated protein kinase (MAPK) pathway specifically in the branching epithelia of developing kidney by genetically abrogating the pathway activity in mice lacking simultaneously dual-specificity protein kinases *Mek1* and *Mek2*. Our data show that MAPK pathway is heterogeneously activated in the subset of G1- and S-phase epithelial cells, and its tissue-specific deletion results in severe renal hypodysplasia. Consequently to the deletion of *Mek1/2*, the activation of ERK1/2 in the epithelium is lost and normal branching pattern in mutant kidneys is substituted with elongation-only phenotype, in which the epithelium is largely unable to form novel branches and complex three-dimensional patterns, but able to grow without primary defects in mitosis. Cellular characterization of double mutant epithelium showed increased E-cadherin at the cell surfaces with its particular accumulation at baso-lateral locations. This indicates changes in cellular adhesion, which were revealed by electron microscopic analysis demonstrating intercellular gaps and increased extracellular space in double mutant epithelium. When challenged to form monolayer cultures, the mutant epithelial cells were impaired in spreading and displayed strong focal adhesions in addition to spiky E-cadherin. Inhibition of MAPK activity reduced paxillin phosphorylation on serine 83 while remnants of phospho-paxillin, together with another focal adhesion (FA) protein vinculin, were augmented at cell surface contacts. We show that MAPK activity is required for branching morphogenesis, and propose that it promotes cell cycle progression and higher cellular motility through remodeling of cellular adhesions.

## Author Summary

Development of the ureter and collecting ducts of the kidney requires extensive growth and branching of an epithelial tube, the ureteric bud. While many genes that control this process are known, the intracellular signaling pathways that underlie renal morphogenesis remain poorly understood. The cellular changes that contribute to ureteric bud morphogenesis, such as adhesion and movements, are guided by intracellular signaling triggered by stimuli at the cell surface. Mitogen-activated protein kinase (MAPK) pathway is known to regulate proliferation in general, but its precise functions during different cell cycle phases are debatable. Moreover, the role of MAPK activity in control of cell adhesion has been demonstrated in cultured cells, but such regulation *in vivo* remains to be elucidated. Here, we examine the importance of the MAPK activity in ureteric bud branching, and find that simultaneous lack of *Mek1* and *Mek2* genes allows elongation of the bud but specifically arrests new branch formation. We show that lack of MAPK activity leads to changes in focal adhesion molecules and E-cadherin mediated cell adhesion and delay in cell cycle progression. Our findings may help to understand the origins of certain congenital malformations in humans.

## Introduction

Receptor tyrosine kinase (RTK) signaling is a key mechanism through which extracellular stimuli guide development of the kidney and many other organs, but the specific *in vivo* functions of intracellular cascades activated downstream of RTKs remain poorly characterized. The kidney develops as a result of classical reciprocal inductive tissue interactions between the nephron-producing metanephric mesenchyme (MM), and the branching epithelium of the ureteric bud (UB), a structure later giving rise to the collecting duct system of the functional organ [1]. Renal differentiation begins with the formation of UB, which invades the surrounding MM, and subsequently starts its branching. UB morphogenesis is largely instructed by the MM, which secretes growth factors such as glial cell-line derived neurotrophic factor (GDNF) and members of fibroblast growth factor (FGF) family. Their RTK receptors, namely RET and FGF receptor 2, expressed in UB epithelial cells, regulate UB development [2].

Based on genetic and *in vitro* experiments, GDNF/RET signaling is required for early UB morphogenesis [3]–[5], while the requirement for FGFR signaling appears to arise later during normal kidney development [6], or in situations where RET signaling is absent [7]. Although the molecular basis of UB branching has been extensively studied, relatively little is known of the cellular cascades and responses regulating the formation of new branches *in vivo*. Binding of GDNF and FGF to their receptors activates several intracellular pathways of which phosphoinositide 3-kinase (PI3K)/AKT, mitogen-activated protein kinase (MAPK) and phospholipase Cγ (PLCγ) function during renal differentiation [8]. Inhibition of the PI3K pathway in kidney organ cultures suggests that primary UB formation depends on chemotactic cell motility induced by this pathway [9], whereas similar experiments with MEK inhibitors suggest that the MAPK pathway is also required for UB morphogenesis [10], [11]. Attempts to genetically confirm such functions are largely missing although deletion of the protein tyrosine phosphatase *Pntpn11*, which positively regulates MAPK, JAK/Stat and PI3K/Akt, suggests that these intracellular cascades also mediate pivotal functions during *in vivo* development [12], [13]. Mutations in specific RET docking sites known to activate certain intracellular pathways indicate that induction of PLCγ via Y1015 as well as simultaneous activation of PI3K and MAPK via Y1062 pathways are involved in renal differentiation [14]–[17].

Active cell proliferation occurs in UB tips [18], which are the major sites for generation of new branches formed through bifurcation of an existing buds [11]. In addition to proliferation, which appears to involve transient delamination of the cells from monolayer [19], active cell movements needing constant turnover of cellular adhesions have been implicated in UB morphogenesis [20], [21]. MAPK pathway, which is well known cell cycle regulator, functions through the RAS-RAF-MEK-ERK cascade, but its specific requirements during different cell cycle phases are highly cell type specific. The activation of RAF kinases leads to rather linear signal transduction upon phosphorylation of dual-specificity protein kinases MEK1 and −2, which in turn phosphorylate ERK1 and −2 (presently their only known substrates) [22]. ERKs have a wide variety of nuclear and cytosolic targets including cyclin D1 and focal adhesion (FA) scaffold protein paxillin, which also associates with MEK [23], [24]. Either disruption of ERK/paxillin complex or lack of ERK induced phosphorylation on serine 83 abolishes cell spreading and branching morphogenesis [24], [25]. Interestingly, paxillin and another FA protein, vinculin, are found also in adherens junctions (AJ), where they associate with β-catenin to modulate adhesion at sites of cell-cell contact [26], [27]. Vinculin stabilizes E-cadherin at AJs where it potentiates E-cadherin mechanosensory responses [28], [29].

Here we have studied the *in vivo* functions of MAPK pathway during renal branching by deleting *Mek1* [30] specifically in UB epithelium in *Mek2* -null background [31]. As previously suggested by chemical inhibition of MAPK in whole kidney cultures [10], [11], our results show definitively that loss of MAPK activity specifically in the UB prevents the generation of new branches while allowing bud elongation. The MAPK pathway appears to contribute to UB branching guidance by carrying out dual functions; it regulates G1/S-phase transition during cell cycle progression, and epithelial cell adhesion through paxillin phosphorylation affecting FA and AJ dynamics.

## Results

### MAPK pathway activity in UB epithelium of developing kidney correlates with S and G phases of the cell cycle

**Figure 1:**
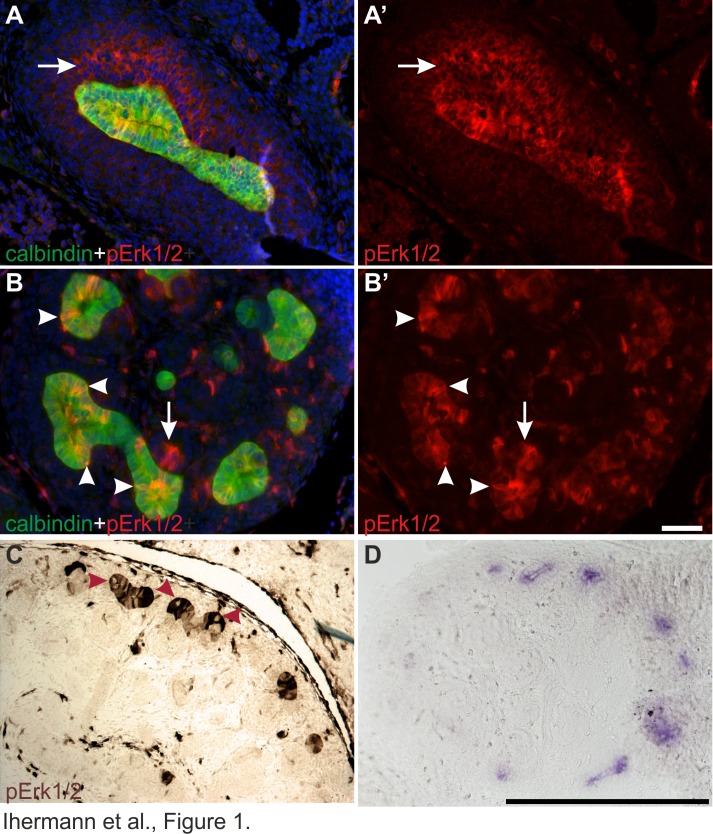
Localization of MAPK pathway activity in developing kidney. (A–A′) Representative cross sections of wild type E11.5 kidneys stained with anti-phospho-ERK1/2 (red) demonstrate MAPK pathway activity both in the ureteric (UB) bud, visualized by calbindin staining (green), and metanephric mesenchyme (arrow). (B–B′) In E13.5 wild type kidneys, pERK1/2 localization in the ureteric bud epithelium is concentrated to UB tips where the staining is unevenly distributed among the epithelial cells (arrowheads), which all express UB marker calbindin (green). Additional pERK1/2 staining is detected in nephron primordia (arrows). (C) Chromogenic pERK1/2 staining on E14.5 wild type kidneys shows strong but heterogeneous MAPK activity in UB tips with lack of activity in sporadic cells (red arrowheads). (D) *Ret* expression in the ureteric bud tips of E13.5 wild type kidney as detected by *in situ* hybridization of mRNA on vibratome sections. In A and B, nuclei are labeled with Hoechst. Scale bars: A–B 50 µm, C–D 500 µm.

The pattern of MAPK pathway activity was first studied in kidneys at different developmental stages. As shown before [20], pERK1/2 localized on one side of Wolffian duct epithelium at E10.5, just before UB outgrowth. A day later when the UB had branched once to form the so-called T-bud, prominent pErk1/2 staining was detected both in the epithelium and surrounding MM (Figure 1A). During subsequent branching, MAPK activity was restricted to UB tip regions, in a pattern similar to *Ret* expression, and to early nephron progenitors in the MM and dispersed cells in the medulla (Figure 1B–D).

**Figure 2:**
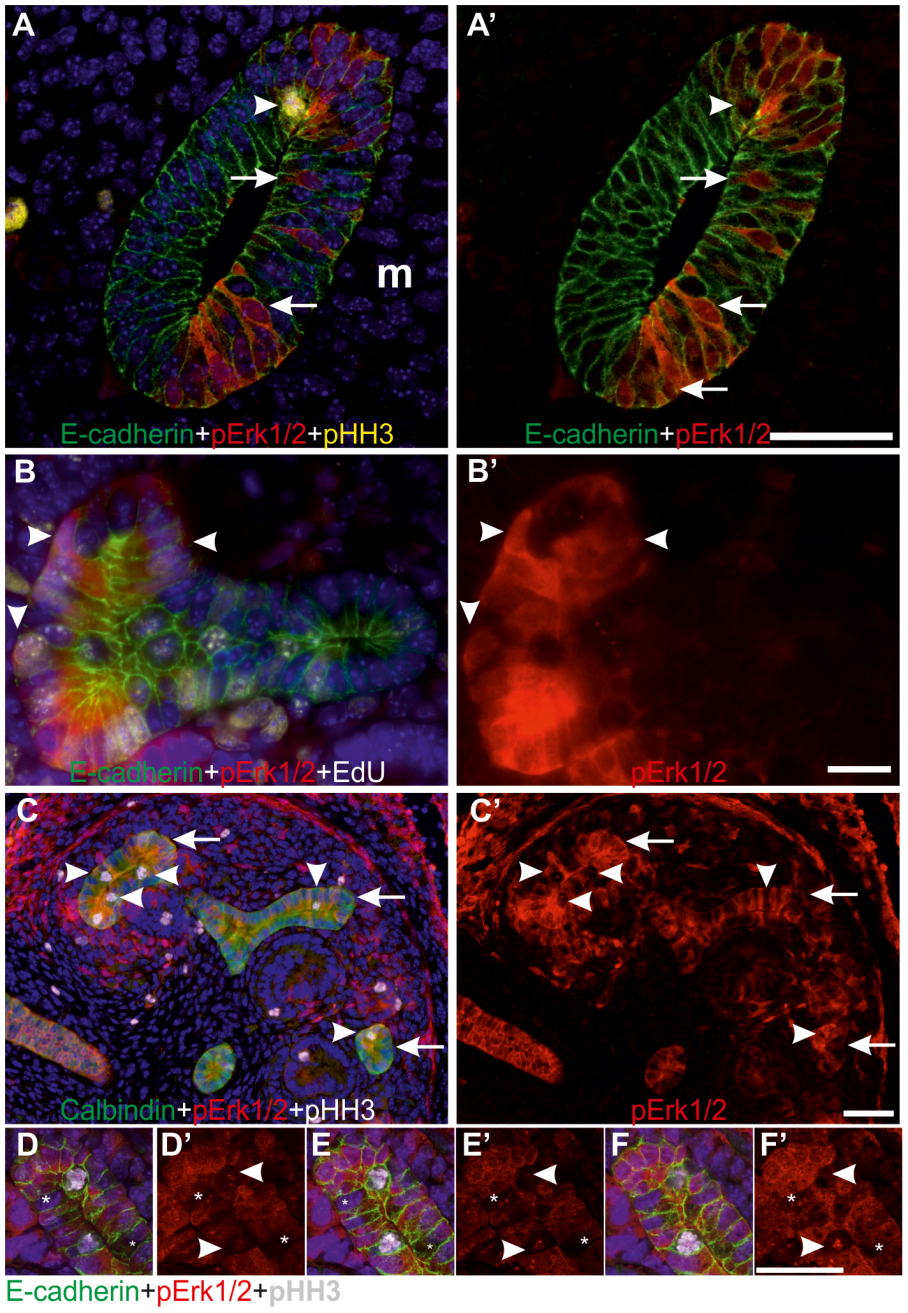
MAPK co-localizes with G1/S-phase markers in the ureteric bud epithelial cells. (A–A′) Confocal images of the wild type Wolffian duct at E10.5, including the region that will give rise to the UB, demonstrate MAPK activity (red) only on the side where kidney mesenchyme is located (m). No pERK1/2 staining was detected in the mesenchyme at this stage. The presumptive UB shows a typical appearance of pseudo-stratified epithelium where cell nuclei (blue) are located at several levels of epithelium stained with E-cadherin (green). Scattered MAPK activity (red) localizes to both cytoplasm and nuclei, where it is detected at different levels (arrows) of epithelium (E-cadherin, green). Mitotic, pHH3-positive (yellow) nuclei (arrowhead) located in apical surface of the epithelium shows no pERK1/2. (B–B′) MAPK pathway is active (red) during and after DNA replication as seen by pERK1/2 staining in some UB epithelial cells that have incorporated EdU (white in B, arrowheads) during an hour pulse. E-cadherin (green) marks cell borders and Hoechst (blue) visualizes nuclei while B′ shows only pERK1/2. (C) MAPK pathway activity (red) and pHH3 (white) in UB tips of E13.5 wild type kidneys. Arrowheads mark the mitotic epithelial cells with pHH3 label, which lack MAPK activity as seen in C′. Arrows indicate UB tips. (D–F′) Confocal stack through E13.5 wild type UB tip shows pERK1/2 (red) in cytoplasm and nuclei of tip cells but lacking in mitotic, pHH3+ (white, arrowheads in C, D′, E′ and F′) and some other cells (asterisks). Scale bars: A&C 50 µm, B, D–F 20 µm.

Closer examination of pERK1/2 staining revealed striking heterogeneity in MAPK activity between adjacent epithelial cells (Figures 1A–B and 2A–F). In the pseudo-stratified E10.5 Wolffian duct epithelium [20], the mitotic nuclei localize at the apical surface, while the S phase nuclei are found on the basal surface, and G1/2 nuclei within the middle epithelial zone (due to interkinetic nuclear migration). Most pERK1/2 positive cells in E10.5 Wolffian duct epithelium were found in the middle and basal zones (Figure 2A). Later, during the active UB branching phase, a similar pattern of pERK1/2 was maintained (Figure 2B–F) suggesting that MAPK pathway is activated in a subset of cells in G- and S-phases of the cell cycle. Accordingly, pulse labelling of proliferating cells with uridine analog 5-ethynyluridine (EdU) followed by simultaneous detection of pERK1/2 and EdU-positive cells showed co-localization in the UB tips (Figure 2B). Notably, while a large fraction of cells in G- or S-phases were positive for pERK1/2, none of the mitotic cells (identified by their round shape and expression of phosphorylated histone H3) were pERK1/2 positive (Figure 2C–F). This was constantly found in six distinct kidneys (E10.5–13.5) accounting approximately 100 UBs, and suggests lack of MAPK activity during mitosis in UB epithelial cells.

### *Mek1* and *-2* functions are redundant in UB morphogenesis

**Figure 3:**
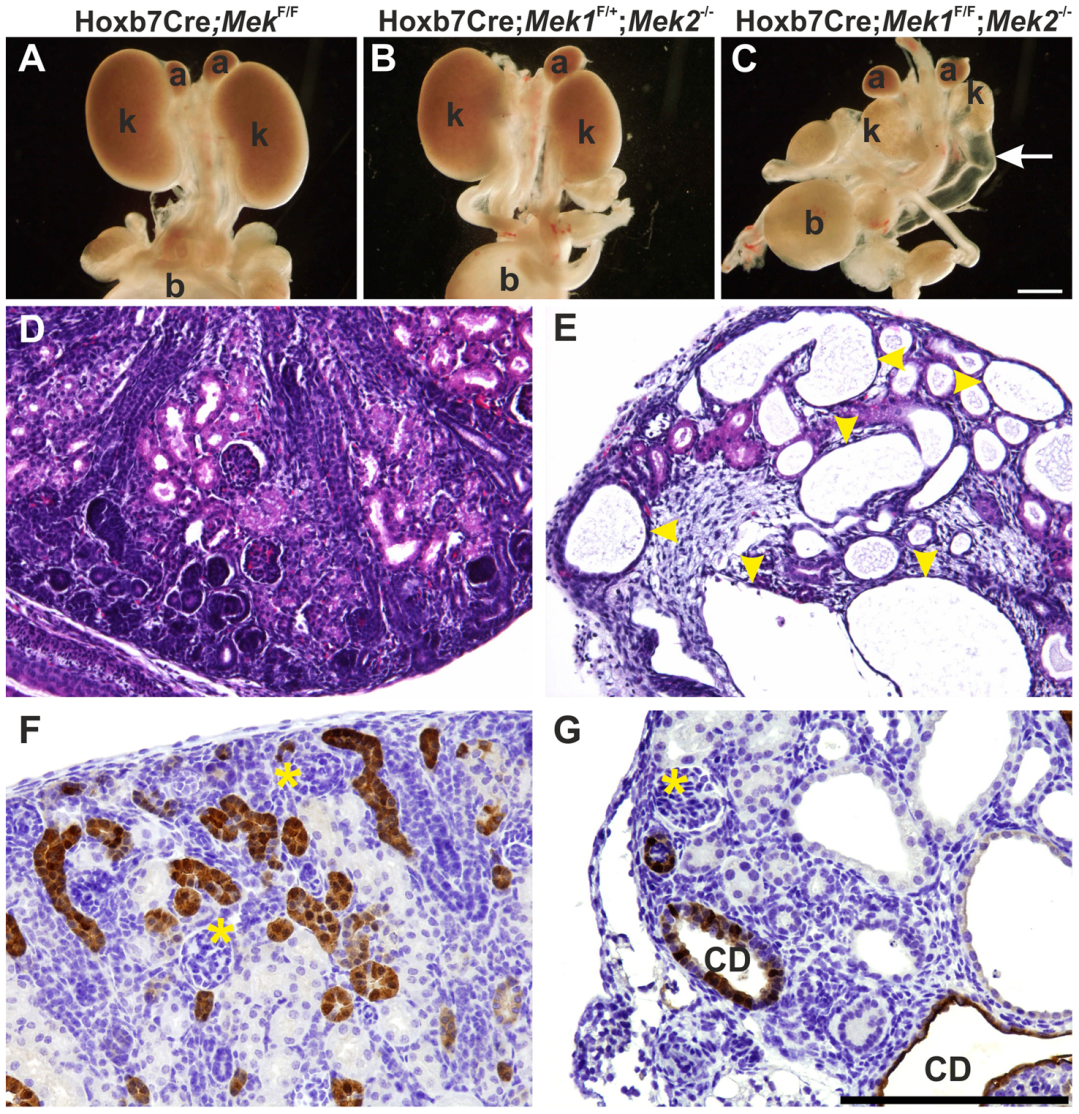
Genetic ablation of MAPK pathway specifically in ureteric bud epithelium results in severe renal hypodysplasia. (A) Newborn kidneys with *Mek1* deleted specifically in ureteric bud (UB) derivatives are comparable to wild type kidneys. (B) Absence of three out of four *Mek1* and Mek*2* alleles is enough to support normal kidney development as shown in newborn Hoxb7CreGFP;*Mek1^F/+^*;*Mek2^-/-^* kidneys. (C) Simultaneous lack of *Mek1* and *Mek2* in UB results in very small kidneys with hydroureters (arrow). Histology of newborn (D) control and (E) dko kidneys demonstrates huge cysts (yellow arrowheads) and reduced nephron epithelium in double knockout newborns. Calbindin staining visualizes numerous UBs and collecting ducts in (F) newborn control kidney whereas (G) the amount of collecting duct epithelia is minimal and dilated in dko kidneys. Asterisks mark glomeruli; a, adrenal gland; b, bladder; CD, collecting duct; k, kidney. Scale bars: A–C 1 mm, D–G 100 µm.

Abundant pERK1/2 in developing kidney (Figures 1 and 2) together with *in vitro* chemical inhibition studies [10], [11] suggested that MAPK pathway could be important for kidney morphogenesis *in vivo*. Conventional knockout of *Mek1* is embryonic lethal while generation of a conditional allele allows its tissue-specific deletion [30] from Wolffian duct and UB lineages using Hoxb7CreGFP transgenic mice [6]. This resulted in normal looking embryonic kidney (Figure 3A and S1A), similarly to *Mek1* deletion in epidermal keratinocytes [32]. Since ubiquitously expressed *Mek2* can phosphorylate ERK1/2 and may compensate the loss of *Mek1* in Hoxb7CreGFP;*Mek1^F/F^* kidneys, we reduced the gene dosages of *Mek1* and *−2* in UB epithelium. Conventional deletion of *Mek2* alone, which results in phenotypically normal mice [31], or UB-specific double heterozygosity for *Mek1* and *–2*, had no effect on UB branching, kidney differentiation or phosphorylation of ERK1/2 (Figure S1D–F and data not show). Normal UB branching pattern and renal differentiation were observed *in vivo* and in organ culture, even in the absence of three out of four *Mek1* and *−2* alleles regardless of allelic combinations (Figures 3B and S1A–L).

### Novel branch formation during UB morphogenesis depends on MAPK activity

Next *Mek1* was removed from UB in *Mek2^-/-^* background to examine effects on renal differentiation. Hoxb7CreGFP;*Mek1* ^F/F^;*Mek2* ^-/-^ (henceforth called “dko” for double knock-out) mice were born in the expected Mendelian ratio (data not shown) but died within 72 h due to obvious renal defects, including severe renal hypodysplasia and sporadic hydroureters (16%) (Figure 3C, E and G). Histological examination showed disorganized medulla-cortex compartmentalization and few but well-differentiated glomeruli with associated tubuli and dilated epithelium (Figure 3D–E). Staining with the collecting duct epithelium marker calbindin and nephron segment markers Tamm-Horsefall and Na/K ATPase indicated that the cysts originate both in collecting ducts and secondarily in nephron tubules (Figure 3F–G and S1M–N).

**Figure 4:**
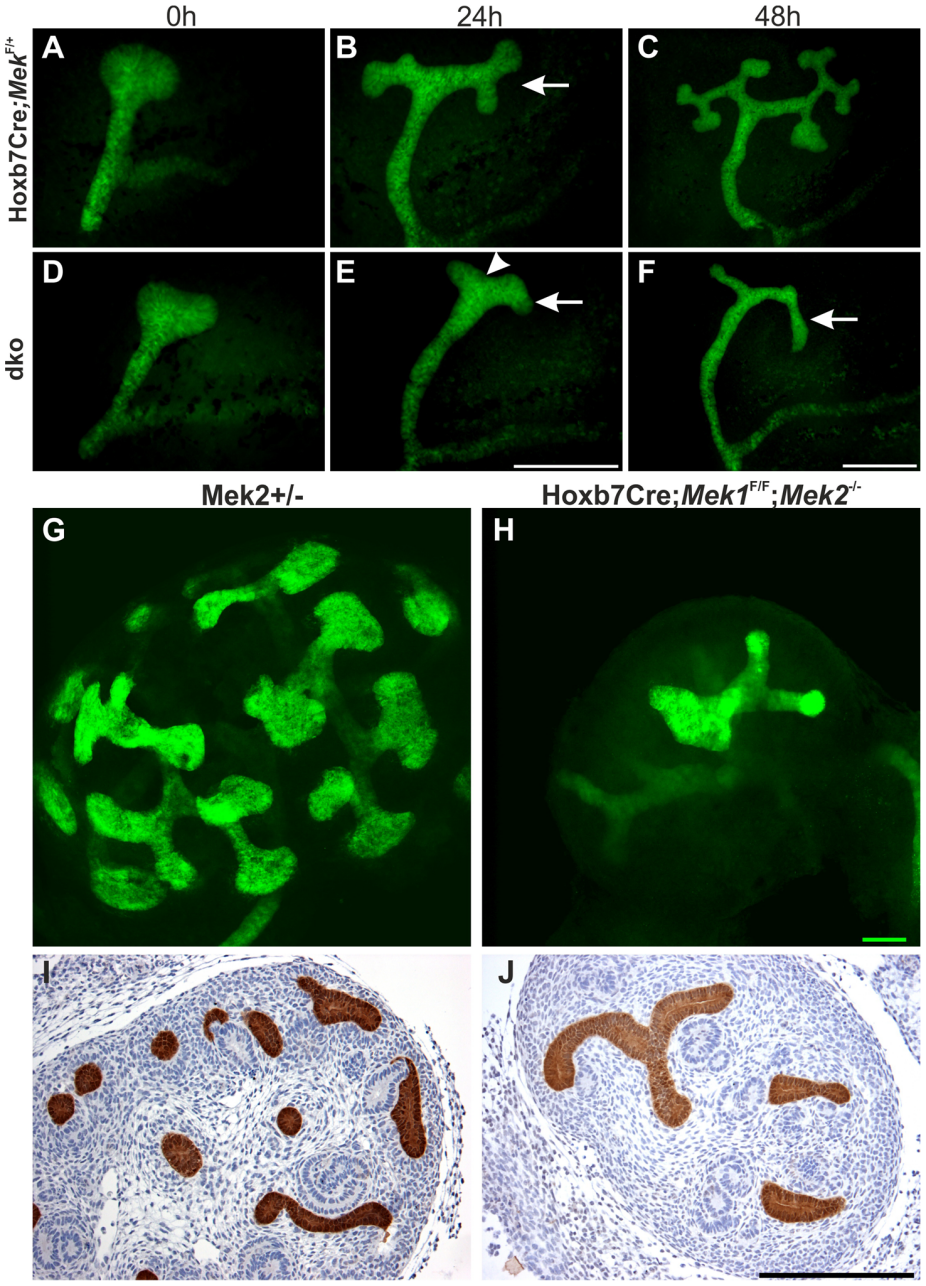
Normal ureteric bud outgrowth in dko kidneys is followed by severely compromised branching morphogenesis. (A–F) Time lapse imaging of kidneys cultured for 48 h, and ureteric buds visualized by the green fluorescent protein tag encoded by the Hoxb7CreGFP construct. (A) In control kidney, ureteric bud is formed and swollen at E11.5, followed by branching (arrow) (B) 24 h later. (C) At 48 h, several generations of branches (average UB tip number: 10.5, n = 6) have been generated in the control kidney. (D) The UB formation in dko kidney at E11.5 is indistinguishable from the control kidney. (E) At 24 h, branching morphogenesis is progressing very slowly in the dko kidney as indicated by deepening of the cleft (arrowhead) on top of the developing T-bud, which however fails to complete and further generate secondary branches (arrows, average tip number: 3.8, n = 5) even at 48 h (F). (G–H) E13.5 ureteric epithelium visualized by calbindin (green) staining, followed by 3-dimensional reconstruction from confocal optical sections, shows the typical shape and distribution of UB tips at the surface of control kidney (G). Note that the control UBs are distributed over the entire surface area of the kidney, while in (H) dko kidney UB tips are very infrequent due to defective branch formation, leaving large areas of the kidney surface devoid of UB epithelium. (I–J) Calbindin staining on E13.5 kidneys shows several UB tips and branches (brown) in (I) control but only few in (J) dko. Scale bar: Scale bars: A–F 500 µm, G–H 50 µm, I–J 250 µm.

Rudimentary kidneys in dko mice suggested that UB branching, the key process by which the kidney grows in size and acquires its typical shape could be perturbed. Time lapse imaging of *in vitro* cultured kidneys demonstrated a remarkable reduction in formation of novel branches in UB tips; average of 10.5 tips in controls was reduced to 3.8 in dko kidneys (Figure 4A–F, p<0.001 two-tailed T-test, n = 5). After generation of the primary UB at the correct time and with normal morphology, the subsequent epithelial morphogenesis in dko kidneys failed to start, and the UB tips usually elongated in only one direction (mean trunk lengths in controls: 142.3 µm, n = 83, three distinct kidneys, and in dko: 195.9 µm, n = 18, two distinct kidneys, two-tailed T-test, p<0.05; compare Figure 4A-C to D–F, S1O). A similar phenomenon was observed in intact dko kidneys imaged by confocal microscopy (Figure 4G–H). Typically very few if any UB tips at E13.5 had enlarged into T-bud resembling structures, which are signs of active branching. Thin UB tips were sparsely distributed in dko kidneys (average of 8.9 tips/kidney), leaving large areas of kidney devoid of UB branches, while in control kidneys UB epithelium was distributed over the entire cortical surface areas of kidneys (average of 41.9 tips/kidney, Figure 4G–I).

Formation of the primary UB at E11.5 in dko kidneys (Figure 4D) was surprising given the strong pERK1/2 staining in early kidneys (Figures 2A and 1A). This suggested that *Mek1* might not yet be deleted by Hoxb7CreGFP in early UB epithelium, or that residual MAPK activity is maintained during the initiation of renal development. MEK1 was ubiquitously expressed in developing control kidneys while specifically lost from the UB of dko kidney from E11.5 (n = 5) onwards (Figure S2A–B), in contrast to pERK1/2, whose localization and staining intensities in dko kidneys were comparable to control kidneys at E10.5 (n = 5, data not shown) and E11.5 (n = 4, Figure S2C–D), and only abolished from the UBs at E12.5 onwards (Figure S2E–F and data not shown).

Application of exogenous GDNF to kidney cultures induces extra UB formation and swelling of UB tips (Figure S2H and [5]). We employed exogenous GDNF and chemical MEK-inhibition in kidney cultures to further test if the MAPK pathway is dispensable for UB outgrowth from the Wolffian duct. MEK-inhibition by UO126 dose-dependently blocked UB branching in kidney cultures (Figure S2I and K) mimicking the defects seen in dko kidneys (Figure 4D–F) and previous findings [10], [11]. Pretreatment with UO126 followed by application of GDNF in the presence of inhibitor blocked typical GDNF responses (Figure S2J), suggesting that the function of MEK1/2 cannot be overcome by RTK activation. Simultaneous UO126-inhibition and activation of RET by exogenous GDNF without UO126 pretreatment (Figure S2L) had the same effect. Thus, normal UB outgrowth in dko kidneys is likely due to delay in abolishing pERK1/2 activity at early stages of renal development.

### MAPK pathway mediates GDNF/RET signaling in regulation of a subset of GDNF target genes

**Figure 5:**
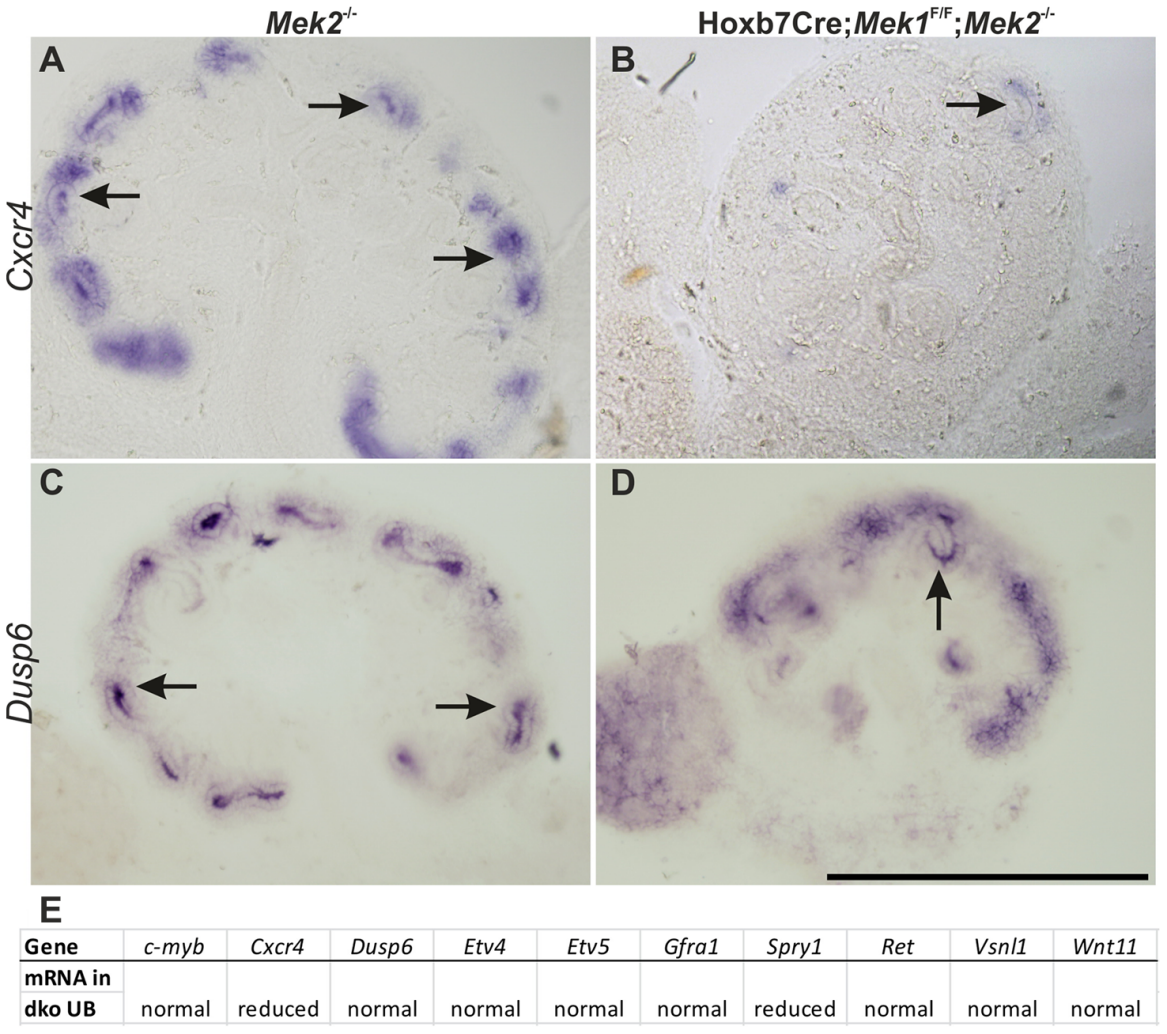
Relationship of intracellular MAPK pathway to RET receptor tyrosine kinase signaling. *In situ* hybridization of the RET signaling target *Cxcr4* in E13.5 (A) control kidney shows expression in UB tips (arrow) as well as in kidney mesenchyme, while expression is lost in (B) UB tip (arrow) of dko kidney. (C–D) Another RET signaling target *Dusp6*, which is a negative regulator of MAPK activity, shows no changes (arrows) in the absence of MAPK pathway activity at E13.5. (E) Table summarizing the expression results of RET signaling targets in UB of dko kidneys. Scale bar: A–D 500 µm.

As GDNF/RET signaling is the key RTK regulating UB morphogenesis in the normal context [7], we wanted to evaluate the linkage between RET and the MAPK pathway at the molecular level. Previous genetic engineering of *Ret* gene on the docking site known to mediate concurrent activation of MAPK and AKT cascades showed their importance for renal differentiation, but evidence for a specific requirement for MAPK activity downstream of RET was lacking [14], [17]. To address this, we examined if known GDNF/Ret signaling targets [33], [34] are regulated through MAPK pathway. *In situ* hybridization of ten GDNF/RET target genes in control and dko kidneys (Figures 5A–E and S3) revealed reduction in chemokine receptor *Cxcr4* and in *Spry1*, a negative regulator of RTK signaling, which exerts its action at least partially by blocking MAPK pathway [35]. Downregulation of specific GDNF target genes in *Mek1/2*-deficient UBs suggested that MAPK pathway is an important intracellular mediator of RET signaling.

### MAPK is required for normal G1/S transition

**Figure 6:**
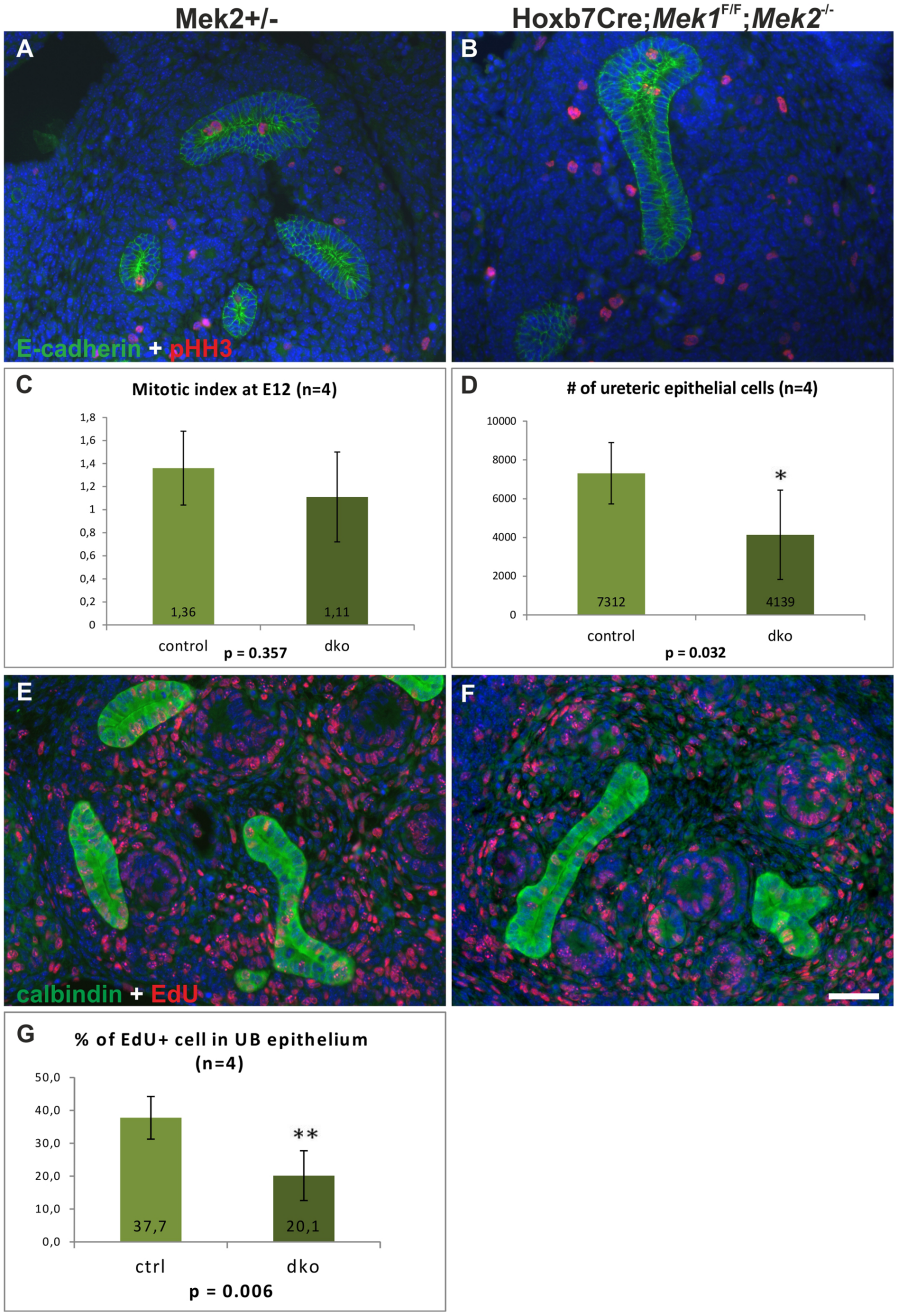
Cell proliferation in the absence of MAPK activity. Examples of E12.5 (A) control and (B) dko kidney stained for pHH3 (red) to highlight cells in mitosis and E-cadherin (green) to visualize the epithelial cell borders. (C) Mitotic indices, calculated as pHH3 positive cells as a percentage of total UB epithelial cells, show no significant difference between the control and dko ureteric tips (n = 4, p = 0.357, 2-tailed T-test, equal variance). (D) The number of total UB epithelial cells in dko kidneys is significantly reduced when comparing to controls, reflecting the overall reduction in the amount of UB branches (n = 4, p<0.05, 1-tailed T-test, equal variance). Examples of E12.5 (E) control and (F) dko kidneys after EdU incorporation (1 h pulse) detected by Click-iT technology (red) and combined with calbindin immunofluorescence staining (green) to identify UB epithelium. (G) Statistical analysis of EdU incorporation in control vs. double mutant UB shows significant decrease in EdU+ cells in mutant UB. Scale bar: 50 µm.

Various studies have shown that RTK signaling can promote proliferation through MEK/ERK pathway [23] and that ERK1/2 regulates G1/S transition in proliferating cells [36], while its function in G2/M transitions and M-phase remains ambiguous [37]–[39]. To reveal the cellular basis of defective UB branch formation in the absence of MAPK activity, we first examined proliferation at the onset of the morphologically distinct phenotype. Analysis of the mitotic indices at E12.5 showed that the percentages of pHH3+ UB epithelial cells were comparable in controls and dkos, but the amount of UB epithelium in dko kidneys was significantly reduced when quantified as total number of epithelial cells (Figure 6A–D). This data indicated that in the absence of *Mek1* and *−2*, UB epithelial cells initially enter mitosis as efficiently as control cells suggesting that G2/M phase occurs independently of pERK1/2. However, at E14.5 dko UB was almost completely devoid of pHH3 (Figure S4A–B) showing a gradual decrease in mitosis. The reduced overall number of mutant UB cells indicated potential problems in cell survival or impairment in other cell cycle phases. Cleaved-caspase3+ apoptotic cells were very sporadically found in UB epithelium of both control and dko kidneys (Figure S4C–D) showing that increased cell death is not causing reduction in cell numbers of dko UB epithelium. Apoptosis was though slightly increased in renal mesenchyme, likely due to decreased UB-numbers in dko kidneys, which leave more mesenchymal cells without induction signal.

The S phase was studied by labeling the newly synthesized DNA with 5-ethynyl-2-deoxyuridine (EdU). Significantly fewer UB cells were EdU+ in the dko epithelium after 1 h pulse, revealing significant reduction of cells in S phase (Figure 6E–G). Since ERK is known to regulate the induction of cyclin D1 [40], [41], whose up-regulation is a key step for G1/S transition, and cyclin D1 at mRNA level is positively regulated by GDNF [33], it was a potential candidate for mediating the effect of the MAPK pathway on the cell cycle. In control kidneys cyclin D1 was up-regulated in early nephron progenitors and throughout the cortical UB epithelium (Figure S4E, G). In the absence of *Mek1/2*, only scattered, single cyclin D1 positive cells were very rarely detected in UB (Figure S4F, H), supporting the idea that MEK1/2-activated ERK functions in the G1/S transition phase during epithelial branching morphogenesis.

### MAPK activity is required for normal cell adhesion

**Figure 7:**
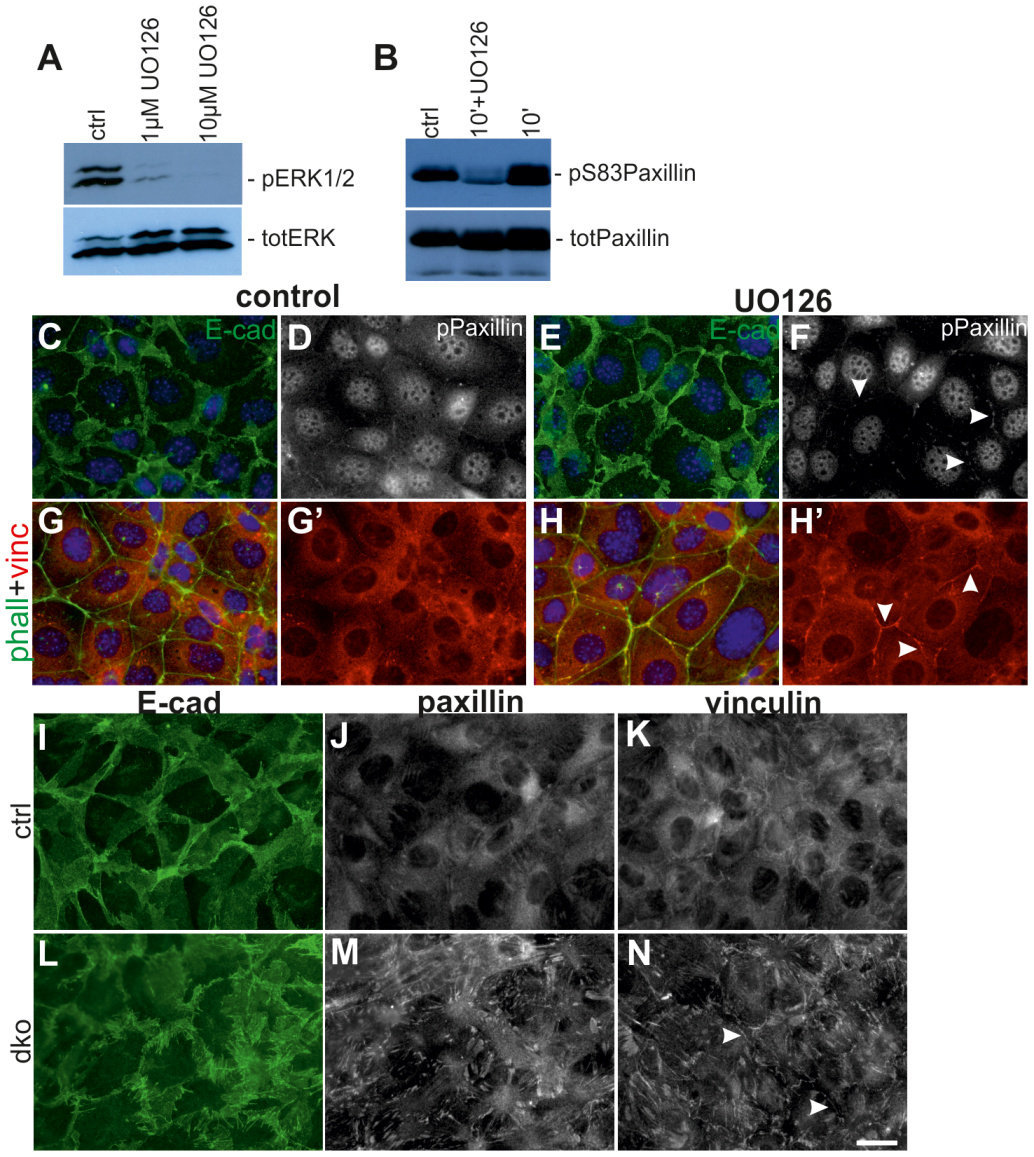
Lack of MEK1/2 function in UB cells intensifies cell surface localization of FA proteins and E-cadherin. (A) Baseline MAPK activity as measured by phosphorylation of ERK1/2 is dose-dependently inhibited by UO126 in 2 h. (B) Paxillin phosphorylation on serine 83 is slightly increased by addition of FBS but greatly reduced by inhibition of MEK1/2 function. (C–D) Control and (E–F) UO126-treated UB cells (15 µM for 4 h) were double-stained with E-cadherin (green), and pPaxillin (white). (C) and (E) show overlay of E-cadherin and nuclei (Hoechst, blue), while pPaxillin is separately shown in (D) and (F). Addition of UO126 increased membrane-associated E-cadherin and FA proteins (arrowheads). (G–H) Co-staining of phalloidin (green) with vinculin (red) demonstrated increase in vinculin at cell-cell contacts upon 4 h of MAPK inhibition. G′ and H′ show only vinculin. (I–N) Primary UB cells originating from E11.5 UBs isolated from (I–K) control and (L–N) dko kidneys. E-cadherin in (I) control cells appears in thick sheet like structures while it is fuzzy and spiky in (L) dko cells. Both paxillin (J, M) and vinculin (K, N) are intensified in FAs of dko cells (M–N) while are more diffusely distributed in control cells (J–K). Additionally to amplified FA localization, vinculin is also intensively distributed to cell surfaces (arrowheads). Scale bar: 20 µm.

Greatly reduced formation of new branches in dko kidneys could involve alterations in cell adhesion properties either at the cell-to-matrix contacts made by focal adhesions, or at the cell-cell contacts formed by E-cadherin based adherens junctions. Since paxillin is a direct phosphorylation (Ser83) target of ERK in innermedullary collecting duct cells [25], we first studied the effect of chemical MEK-inhibition on pPaxillin in a ureteric bud derived cell line [42]. MAPK activity was dose-dependently inhibited by UO126, which also reduced significantly the level of pSer83 paxillin (Figure 7A–B). Immunofluorescence staining showed that inhibition of MAPK activity caused reduction in pPaxillin cytosolic pools but its plasmamembraneous localization was intensified together with E-cadherin, which appeared also stronger at the cell surfaces (Figure 7C–F, S5A–B). Simultaneously vinculin, another FA protein was also more pronounced in the cell surface (Figure 7G–H′). This preferential membranous localization was time dependent as after 2 h of UO126 such differences were not obvious (data not shown).

We next tested if genetic loss of MAPK activity could have changes in FA proteins by generating primary cell cultures from UBs isolated from control and dko kidneys. All control UBs dissected at E11.5–12.5 (n = 10) produced single cell monolayers in approximately 48 h in culture [21], [42], [43], but dko UBs isolated at E12.5 were slower in delaminating from the epithelium (7/7) and two out of seven samples failed to generate monolayers (data not shown). We thus isolated UBs from E11.5 dko kidneys (before pERK1/2 was lost and when the morphological phenotype was still comparable to controls) (Figure S2D, 4C–D), and found that monolayer formation was significantly improved (S5C–D′) but dko cells displayed thick, spiky E-cadherin at cell surfaces (Figure 7I, L). The dko cells remained tightly packed and impaired in spreading after 48 h of culture as seen by very strong and large appearance of FAs (Figure 7J–K, M–N).

**Figure 8:**
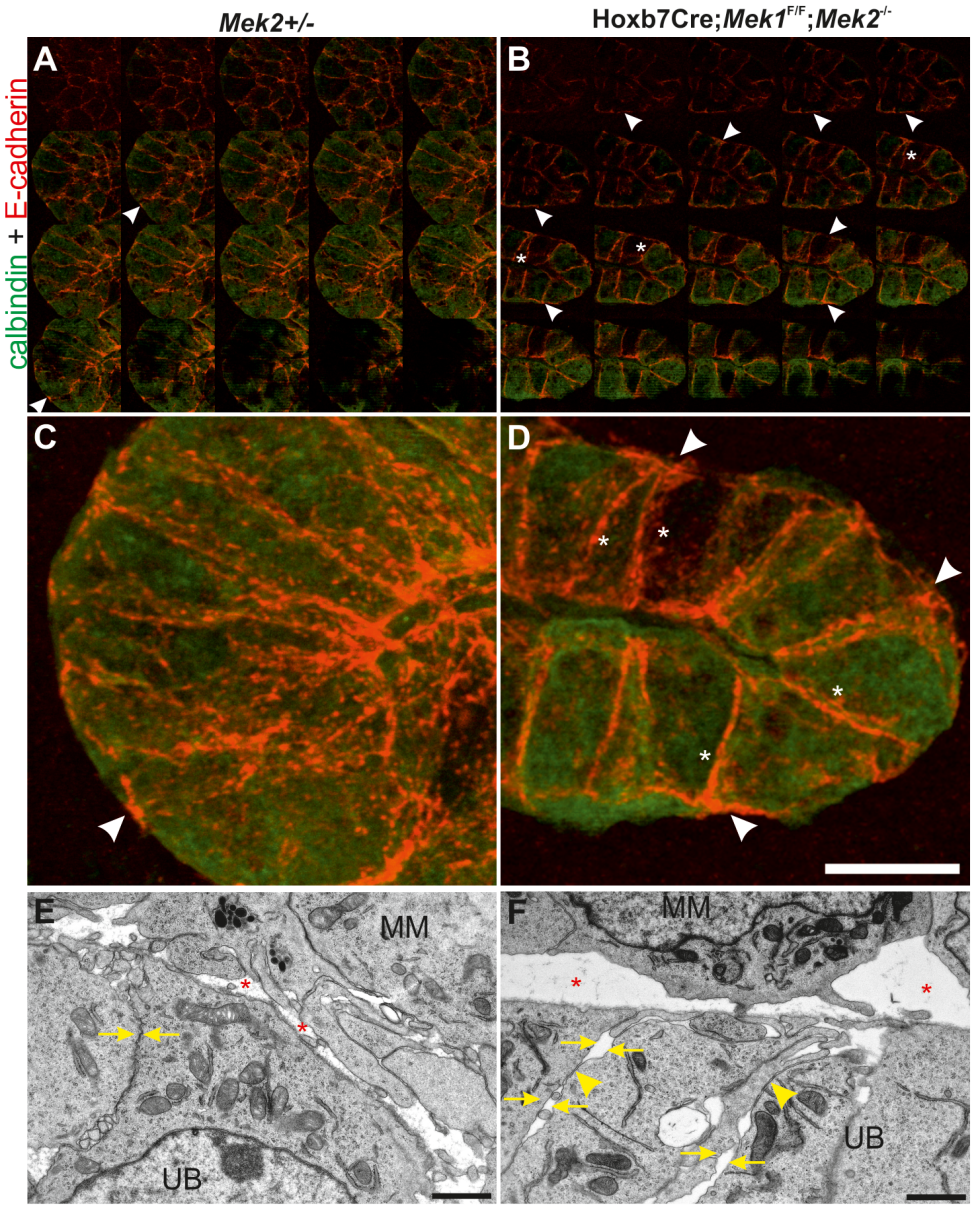
MAPK pathway activity is required for normal E-cadherin localization and epithelial cell adhesion. (A) Merged montage of confocal image through E13.5 control and (B) dko UB stained with calbindin (green) and E-cadherin (red) to show the cell-cell contacts. (C–D) Maximal intensity Z projections of stacks shown in A and B, respectively. Arrowheads point to the baso-lateral points, which show only occasional weak E-cadherin in control UB (A, C), but strong and widespread localization in the UB lacking MAPK activity (B, D). Asterisk indicates stronger intensity at lateral membranes in dko epithelium. (E) Electron microscopy image of E12.5 control UB shows continuous cell-cell contacts (arrows) while (F) in dko UB, the connections are disrupted at several sites (arrows), but the sites where they are maintained (arrowhead) appear electron dense. Also extracellular space (asterisk) between UB epithelial cells and metanephric mesenchymal (MM) cells is enlarged in dko kidneys. Scale bars: C–D 10 µm, E–F 2 µm.

Like FAs, AJs are constantly formed and disassembled during development and tissue homeostasis [44]. We next studied AJ molecule E-cadherin, which localized abundantly in the apical end of lateral cell walls of the control UB tips, being otherwise uniformly distributed along the lateral membranes (Figure 8A, C), while in medullary UB epithelium occasional staining was also observed in the basal end of lateral membranes (arrows in Figure S6A). The UB tips of dko kidneys displayed stronger overall E-cadherin staining than controls (Figure 8B, D, asterisks in S5B). Additionally, E-cadherin localization had shifted from apical to more basal sides of lateral membranes, and was also accumulating in basal membranes (Figure 8B, D) where it was rarely observed in control UB tips. Our visual observations were confirmed by quantitative measurements: E-cadherin intensities in general and the ratios of basal-to-lateral intensities were significantly higher both in cortical and medullary UB epithelium of dko than control kidneys (Figure S6C).

The cytoplasmic domain of E-cadherin is linked to the cytoskeleton through a complex of proteins including p120-, β- and α-catenins [45]. We analyzed β-catenin (Figure S6D–G″) and filamentous actin (Figure S6H and J), which appeared normal in dko kidneys. The function of p120 and related proteins ARVCF and p0071 is to cluster and stabilize E-cadherin at AJs [46], but they appeared normal in dko kidneys (data not shown). While the shift in E-cadherin subcellular localization could also reflect general problems in apical-basal polarity, the apical markers Par3 (data not shown) and ZO1 localized to apical cell surface similarly in control and dko UB epithelium (Figure S6I and K), indicating that apical cell polarity was maintained in the absence of MAPK activity. Staining of control and dko kidneys with tight junction marker claudin7 [47] revealed similar localization in wild type and dko UBs (Figure S6L–M) suggesting that MAPK activity is specifically involved in regulating E-cadherin mediated AJs.

To investigate potential functional consequences of the altered FAs and E-cadherin distribution, we analyzed UB epithelium by electron microscopy (EM). Control UB epithelium appeared as a uniform sheet of cells tightly apposed through cell-cell contacts on their lateral membranes, while the double mutant epithelium had increased extracellular space and many gaps between neighboring cells as seen by disintegration of the lateral membranes at several sites along their contact surface (Figure 8E–F, S7). However, the contact sites where adhesion was maintained appeared more electron dense.

## Discussion

By genetic studies focusing on MAPK pathway functions in renal development, we show that only one allele of either *Mek1* or *Mek2* is enough to support normal renal branching. Simultaneous deletion of both genes abolishes formation of new branches and complex 3D patterns and shows that this pathway is a necessary mediator of RTK signaling. The functional requirement for MEKs in UB morphogenesis is similar to that reported in skin [32] but different than what has been observed in placenta, where double heterozygosity results in embryonic lethality [48]. Similarly, *Erk1*;*Erk2* gene dosage and protein levels are critical for survival and normal proliferation as shown by generation of double heterozygotes, most of whom die during gestation [49]. Such differences in functional requirements of MAPK activity imply tissue specific roles for the pathway components and call for investigations on their specific contributions to development and homeostasis.

While RET is an important RTK regulating renal branching, its exact cellular functions (e.g., proliferation, migration, extracellular matrix remodeling, changes in cell shapes and adhesive properties) and their precise contributions have remained indistinct [50]. Identification of GDNF/RET targets recently revealed several interesting candidates for mediating these functions [33], [34] but the intracellular cascades regulating the expression changes remain to be solved. Similarly to previous chemical inhibition studies [33], we saw that the expression of transcription factors *Etv4* and -*5* was normal whenever UBs were present and thus does not require MAPK activity. This is opposite to the requirement of tyrosine phosphatase Shp2, which appears to regulate *Etv4* and -*5* [12]. The negative RTK regulator, *Spry1*, which suppresses MAPK activity, and chemokine receptor *Cxcr4*, which is involved in migration in several cell types [51] were the only genes examined whose expression was reduced in dko UB epithelium. Our results suggest that different intracellular cascades regulate expression of specific target genes, and further experimentation will reveal potential genetic interactions between RET signaling and MAPK pathway.

We show that lack of MAPK activity disturbs cell proliferation by reducing the number of cells in G1/S without primarily affecting mitosis, which is in line with the finding that pERK1/2 staining was absent in the mitotic, pHH3+ cells. Reduction in markers of G1/S phase but nearly normal cell numbers in M phase suggest changes in cell cycle kinetics, so that the double mutant UB cells spend a longer time in M-phase than control cells. Such a phenomenon was demonstrated in human retinal pigment epithelia, where sustained inhibition of ERK1/2 transiently delays the cell cycle progression [37]. The fact that mutant kidneys are smaller than those in control mice, together with the reduced cell number in G1/S, supports the view that MAPK activity, through the regulation of cyclin D1 levels in UB epithelium, is required for progression from G1- to S-phase as shown previously for several cell types [52]. Consequently the defects in G1/S transition also have an effect on mitosis, which is reduced at later stages of kidney development.

In epithelial remodeling and morphogenesis, during which cells move relative to each other, cell-matrix and cell-cell contacts are constantly assembled and disassembled. We observed that UB epithelium lacking MAPK activity had significantly increased E-cadherin in general and particularly extending to more baso-lateral location than in controls, indicating problems in AJ dynamics. Rapid turnover of the E-cadherin-based homophilic AJs involves its endocytosis and recycling back to the plasma membranes [53], and defects in such processes could result in sustained cell surface localization of E-cadherin. During endocytosis, internalization of E-cadherin is initiated by tyrosine phosphorylation of E-cadherin, which induces its dissociation from p120 [54]. Normal distribution of p120 and related proteins (p0071 and ARVCF) in dko UBs suggests normal E-cadherin endocytosis, as increased localization of p120-family proteins at the cell surface would be expected if endocytosis was affected. However, normal appearance of p120 does not conclusively exclude changes in E-cadherin endocytosis or recycling.

Growing evidence indicates active crosstalk between different adhesion sites and this can be at least partially mediated by localization of certain proteins like vinculin and paxillin to both focal adhesions and AJs [26], [55]. Paxillin is a direct phosphorylation target of ERK-proteins and lack of pERK-induced phosphorylation on serine 83 of paxillin leads to functional defects in cell spreading and branching morphogenesis [24], [25]. We found accordingly that inhibition of MAPK activity in a ureteric bud-derived cell line [42] reduces general pPaxillin levels but also observed a simultaneous shift in its cellular localization to the cell-cell contacts, where concomitant increases in vinculin and E-cadherin levels were also detected. Additionally, primary cells derived from the double mutant UBs were impaired in their capacity to form monolayers, remained tightly packed and displayed stronger FAs than control cells, which all argue that such regulation is functionally significant. Both paxillin and vinculin bind to β-catenin in AJs, where at least vinculin has the potential to stabilize E-cadherin and, in certain cell types, to potentiate mechanosensing [27]–[29]. Taken together with the observation that lack of MAPK activity both *in vivo* and *in vitro* intensifies E-cadherin localization on cell surfaces, and recent evidence for cross-talk between FA and AJ, we suggest that MEK1/2-activated ERK1/2 regulates cellular adhesion by phosphorylating paxillin at Ser83, which facilitates normal FA dynamics and composition. The molecular mechanism by which lack of paxillin phosphorylation leads to increased plasmamembranous localization of itself and vinculin remains a subject of future studies, but non-receptor tyrosine kinases Src and focal adhesion kinase (FAK) as well the small GTPase Rac1 are shown to mediate shuttling between FAs and AJs in other cell types, and therefore are good candidates [55], [56].

Increased E-cadherin in the dko UB epithelium intuitively suggests stronger intercellular adhesion, but electron microscopy revealed big gaps in lateral membranes of adjacent cells. However the contact sites that were maintained in the dko epithelium appeared electron dense, suggesting that they might mediate strong contacts. Therefore it is possible that the gaps in lateral membranes are actually caused by increased adhesion at the localized sites where the contacts are maintained and then such stronger contacts generate ruptures to membranes next adhesion when cells are trying to move relative to each other. In support of this, vinculin in AJs promotes high E-cadherin based adhesion strength [57].

Our findings demonstrate the importance of *Mek1*/*Mek2* in activation of MAPK pathway during UB branching, which is largely blocked in dko kidneys. Based on our results we suggest that MAPK activity regulates cyclin D1-mediated progression of cell cycle from G1 to S, and normal cellular adhesion through phosphorylation of paxillin in FAs. Absence of MAPK activity amplifies FA proteins vinculin and remnants of pPaxillin on cell surfaces where they participate in stabilization of E-cadherin to AJs possibly to strengthen intercellular adhesion. Taken together with heterogeneous pERK1/2 localization in UB epithelium, where certain cells show high ERK-activity and others no activity at all, this may imply that MAPK activity tunes cells for higher motility and thereby together with driving proliferation promotes novel branch formation.

## Materials and Methods

### Mouse strains, organ culture experiments and EdU-pulse labeling

*Mek1*-floxed, *Mek2*-null mice and Hoxb7CreGFP mice and their genotyping by PCR has been described [6], [31], [32]. All mice were on mixed genetic background with contributions from C57BL6/Rcc and 129/SvEv. Embryonic staging was as described earlier [58] and all experiments were approved by Finnish Animal Care and Use Committee.

NMRI or Hoxb7CreGFP mice were used for MEK-inhibition experiments. Kidneys were cultured on Trowell-type system in medium of F12:DMEM/10%FBS/Glutamax/penicillin-streptomycin, and imaged with epifluorescent microscope (see below). UO126 (Cell Signaling technologies, Inc.) dose-dependence testing was done with 5, 10, 15 and 50 µM by adding only the inhibitor, or UO126 in combination with GDNF (produced by Icosagen Ldt., see details in Supplementary information) either at the same time or sequentially. Already 5 and 10 µM concentrations of UO126 inhibited UB branching but upon replacement with GDNF resulted in some degree of response, while 15 µM mimicked closest the UB pattern in dko kidneys.

EdU (25 mg/kg) was injected intraperitoneally to pregnant females at day 12 of pregnancy. After an hour pulse the females were sacrificed and embryos collected for 4% PFA-fixation followed by standard processing for paraffin embedding (see below).

### Immuno- and hematoxylin-eosin (HE) staining

Embryos and tissues of indicated stages were collected and fixed with 4% PFA. Further processing for frozen and paraffin sections was according to standard procedures, the latter utilizing an automatic tissue processor (Leica ASP 200). The primary and secondary antibodies used are shown in Table S1. HE, whole mount and frozen section immunostaining were performed as previously described [20], [58]. Paraffin sections were gradually dehydrated followed by heat induced antigen retrieval. Sections were then blocked with 10% fetal bovine serum (Hyclone) for one hour at RT followed by o/n primary antibody incubation. Fluorescent detection was performed similarly to that with frozen sections and chromogenic detection was done with EnVision Detection System-HRP (DAB) kit (Dako). EdU incorporation detection step was done by using Click-iT EdU Alexa Fluor Imaging kit (Molecular Probes/Invitrogen) according the manufacturer's instructions after the antigen retrieval.

### *In situ* hybridization

PFA-fixed E13 urogenital blocks were embedded in 4% low melting agarose (NuSieve GTG, Lonza) and 50 µm thick vibratome sections were cut (HM650, Microm Int.) for whole mount in situ hybridization performed with InSituPro automate (Intavis) according [59].

### Electron microscopy, imaging and statistical analyses

For EM, E11.5 and E12.5 kidneys were fixed with 2% glutaraldehyde (Fluka) followed by 1% osmiumtetroxide (EMS) post-fixation and graded series of dehydration. Transitional acetone incubation was performed before gradual embedding in Epon (TAAB Ltd.). Ultrathin sections (Leica ultracut UCT ultramicrotome, Leica) were collected on pioloform-coated single-slot copper grids and post stained with uranyl acetate and lead citrate for analyzing with transmission electron microscope (Tecnai G2 Spirit 120 kV TEM with Veleta and Quemesa CCD cameras) operated at 100 kV.

Epifluorescence images were produced with Zeiss Imager M2 Axio (Germany) equipped with Zeiss AxioCam HRm camera (Germany) and Axio Vision 4 software. Confocal imaging was done with mutiphoton Leica TCS SP5 MP confocal microscope (Germany) utilizing LAS-AF software. Chromogenic immunostaining was imaged with Olympus BX61 light microscope (Japan) equipped with Olympus Color View Soft Imaging System camera (Japan) and Cell F imaging software.

Quantification of mitotic indices from total of four control and four dko kidneys sectioned through to give 150 and 127 sections, respectively, and stained similarly as previously described [21] followed by counting of all UB epithelial cells in both groups at 40× magnification. EdU percentages were counted at 40× magnification from entire kidneys sectioned through (n = 4 for both groups). Proliferation significances were tested with Independent samples T-test (1-tailed, equal variances). Quantification of stalk lengths, as indicated in Figure S1O, was performed from 43 trunks of three distinct control kidneys, and 18 trunks from two distinct dko kidneys using Image J program. Statistical testing was done with Independent samples T-test (2-tailed, unequal variances) utilizing the SPSS software. For quantification of basal E-cadherin intensities, E13.5 UBs were imaged with multiphoton confocal microscope at 63× magnification with 251.8 nm optical section thickness. Intensity measurements were performed with Image J on 12-bit images with applied 2 pixel median filter. The average basal and lateral pixel intensities were obtained as mean grey values (MGV) from four tips/genotype so that 70 control cells and 97 dko cells in total were analyzed in the way indicated in Figure S6C′. Basal-to-lateral intensity ratios were calculated by dividing basal MGV with lateral MGV and cortical and medullar UB regions were compared with Independent samples T-test (two tailed, equal variances) employing SPSS program after removing three outlier ratios from the control and one from the dko set.

### Cell culture and western blotting

UB cells were cultured in DMEM/10%FBS/Glutamax/penicillin-streptomycin media. 1×10^5^ cells for MEK-inhibition experiments (2 h) were plated 24 h before performing the inhibition by UO126 after which they were collected in lysis buffer. For paxillin phosphorylation, UB cells were serum-starved for 4 h and followed by fetal bovine serum (FBS) treatment of indicated times and 15 µM UO126 was used for inhibiting MAPK activity 30 min prior plus during the induction with FBS. Western blotting was done as previously described [60]. Rabbit anti-pErk1/2 (Cell Signaling, 1∶2000), anti-ERK2 (K-23, Sant Cruz, 1∶1000), p(S83) paxillin (ECM Biosciences, 1∶1000) and mouse paxillin (ECM Biosciences, 1∶1000) were used with HRP-conjugated secondary antibodies to detect proteins which were visualized by Pierce ECL Western Blotting detection system (Thermo Scientific) and Fuji film LAS1000.

For immunofluorescence staining cells were treated with 15 µM UO126 for 4 h, then fixed with 4% PFA for 10 min and stained with anti-E-cadherin (R&D Systems, 1∶300), pPaxillin, vinculin (Sigma, 1∶500) and 568-phalloidin followed by visualization of primary antibodies with corresponding Alexa-fluor secondary ABs (1∶400, Jackson Immuno Research). Cells were imaged by Zeiss Imager M2 Axio (see above).

### Ethics statement

All work on animals was conducted under PHS guidelines and approved by the relevant Institutional Animal Care and Use Committees.

## Supporting Information

Figure S1 Ureteric bud branching in the absence of, or with reduced, MAPK pathway activity. (A) Deletion of *Mek1* alone specifically in the UB epithelium has no effect on renal development as seen by the normal morphology and branching pattern in E13.5 Hoxb7CreGFP;*Mek1^F/F^* kidney, where the UB is visualized by the GFP. (B) Deletion of three out of four *Mek1* and *-2* alleles (in any combination) is enough to support normal growth and branching of the UB as shown by GFP in E13.5 Hoxb7CreGFP;*Mek1^F/+^;Mek2^-/-^* kidney. (C) Severely reduced UB branching morphogenesis in the absence of MAPK activity is illustrated by GFP-tag in UB of dko kidney. (D–L) *In vitro* organ culture of (D–F) control kidneys and (G–L) those lacking three out of four *Mek1* and *-2* alleles show no differences in their capacity to form new branches during the 48 h observation period. (M) Na/K ATPase and (N) Tamm-Horsefall staining in newborn dko kidney. (O) Illustration of stalk length measurements from E11.5 wild-type kidneys cultured for 48 h where the length of every UB stalk is measured from one branch point to the next. Scale bars 500 µm.
(TIF)

Figure S2 MAPK pathway activity and formation of primary ureteric bud. (A) MEK1 is ubiquitously distributed in E11.5 control kidney, while it is missing from most UB epithelial cells in (B) dko UB. (C) Prominent MAPK activity detected by pERK1/2 antibody staining (red) is observed in early ureteric bud (arrows) formed in E11.5 control embryo (n = 4). (D) MAPK activity (red) is maintained in UB epithelium (arrows) of dko kidney at E11.5 (n = 5). (E) The pattern of pERK1/2 staining in E13.5 control kidney demonstrates MAPK pathway activity in UB tips (arrows) and freshly induced nephron primordia (asterisk). (F) The double knockout UB tip (arrow) is depleted of pERK1/2 staining (red) although it is maintained in newly induced nephron primordia (asterisk). Insets in (C–F) show the corresponding sections labeled with UB epithelial marker calbindin (green). (G) E11.5 control kidney cultured for 48 h and stained with calbindin (green) to visualize UB epithelium shows renal-type branching pattern, which is sustained (H) in the presence of exogenous GDNF (100 ng/ml). Extra GDNF causes typical dilatation of UB tips (asterisk) and ectopic bud formation from the nephric duct (arrows) mimicking the normal bud formation in the earliest stage of kidney development. (I) Chemical inhibition of MAPK pathway with low (5 µM) concentration of UO126 in cultured E11.5 kidney disturbs the normal branching of the UB. (J) Pretreatment of E11.5 kidneys with 5 µM UO126 followed by addition of GDNF (100 ng/ml) in the presence of MEK-inhibitor (5 µM) inhibits normal branching and typical response to extra GDNF. (K) Inhibition of MEK1/2 with 15 µM UO126 blocked formation of new UB branches. (L) Simultaneous inhibition of MAPK pathway and GDNF stimulus blocks the normal effects of exogenous GDNF. Scale bars: A–D 100 µm, E–J 1 mm.
(TIF)

Figure S3 Expression of GDNF target genes in UB epithelium. E13.5 vibratome sections of (A–F) control and (G–L) dko kidneys were hybridized with (A, G) *c-myb*, (B, H) *Etv5*, (C, I) *Gfrα1*, (D, J) *Spry1*, (E, K) *Vsnl1* and (F, L) *Wnt11*. Scale bar: 500 µm.
(TIF)

Figure S4 Apoptosis and proliferation in the absence of MAPK pathway activity. (A) E14.5 control and (B) dko kidneys stained with pHH3 (white), cleaved-Casp3 (red) and UB-marker calbindin (green). UB epithelial cells in mitosis are indicated by arrows. (C) E12.5 control and (D) dko kidneys stained with markers of cell death (cleaved-Casp3, red), proliferation (pHH3, white) and epithelium (E-cadherin, green) show comparable patterns of apoptotic and mitotic nuclei. (E) ERK target cyclin D1 (red), which is required for G1/S transition during the cell cycle progress, is found in differentiating nephron primordia (asterisk) and in most of the UB epithelial cells (green) of E12.5 control kidney. (F) The vast majority of UB cells (green) lack cyclin D1 in dko kidneys while its pattern in nephron primordia (asterisk) remains normal. (G–H) Images showing only cyclin D1 staining seen in E and F, respectively. Arrows indicate the UB epithelium. Scale bar: 50 µm.
(TIF)

Figure S5 Low magnification images of those shown at higher magnification in Figure 7C–F. (A) control and (B) UO126-treated UB cells stained for E-cadherin (green) and F-actin visualized by Alexa568-conjugated phalloidin (red) show more E-cadherin on cell membranes. (C–D) Ureteric buds isolated from E11.5 (C) control and (D) dko kidneys cultured for 48 h (C′ and D′, respectively) to set-up monolayer cultures. Scale bars: 50 µm.
(TIF)

Figure S6 Adherens junction changes appear specific to E-cadherin. Merged confocal image of E13.5 (A) control and (B) dko kidneys stained with calbindin (red) to illustrate ureteric epithelium, and E-cadherin (green) to show the cell-cell contacts. Arrows point to the medullary, more mature UB with stronger E-cad intensity, asterisk point UB tips. (C) Average basal-to-lateral ratios of E-cadherin intensities in control (blue and green) and dko (light blue and light green) UB epithelial cells. In the inset C′, the corresponding cell membranes are indicated in blue and green, respectively. The intensities in cortical and medullary UB epithelium are stronger in the absence of MAPK activtity (*p<0.05 and **p<0.01, respectively, 2-tailed T-test, n = 70 cells in seven ctrl UBs, and 97 cells in 11 dko UBs). (D) β-catenin (green) localization in E13.5 control and (E) dko UBs (calbindin, red). Insets in D and E show the cross section of entire kidney at E13.5, and boxed area is enlarged in the actual image. Confocal stack images of β-catenin localization in cell membranes of (F–F″) control and (G–G″) dko UB. (H) Phalloidin (red) is distributed to apical and basal membranes of UB epithelium and shows very little if any co-localization with E-cadherin (green) in basal membranes of E13.5 control UB epithelium. (I) Double staining of E-cadherin (green) and apical cell polarity marker ZO1 (purple) in E13.5 UB of control kidney. (J) Several sites of co-localization (yellow) and domination of green signal (arrows) demonstrate E-cadherin localization on basal membrane of UB deficient for MAPK pathway. (K) UB epithelium of E13.5 dko kidneys show no changes in apical polarization (ZO1, purple), while demonstrating the mis-localization of E-cadherin to basal membranes in mutant epithelium (arrows). (L) Another junction protein, Claudin-7 (red), is co-expressed with E-cadherin (green) in medullary regions (asterisks) of control UB and (M) MAPK pathway deficient UB. ZO1 is shown in purple, arrows point to the UB tips. Scale bars: 50 µm.
(TIF)

Figure S7 Low magnification electron microscopy (EM) image of E12.5 (A) control and (B) dko kidneys, where yellow rectangles indicate the regions of UBs magnified in Figure 8E–F, respectively. Scale bars: 50 µm.
(TIF)

Table S1 Primary and secondary antibodies used for immunochemistry and biochemistry.
(XLSX)

## References

[1] Saxen L (1987) Organogenesis of the Kidney. Cambridge: Cambridge University Press.

[2] Costantini F, Kopan R (2010) Patterning a complex organ: branching morphogenesis and nephron segmentation in kidney development. Dev Cell 18: 698–712. 20493806 10.1016/j.devcel.2010.04.008 PMC2883254

[3] Costantini F (2012) Genetic controls and cellular behaviors in branching morphogenesis of the renal collecting system. Wiley Interdiscip Rev Dev Biol 1: 693–713. 22942910 10.1002/wdev.52 PMC3430146

[4] Schuchardt A, D'Agati V, Larsson-Blomberg L, Costantini F, Pachnis V (1994) Defects in the kidney and enteric nervous system of mice lacking the tyrosine kinase receptor Ret. Nature 367: 380–383. 8114940 10.1038/367380a0

[5] Sainio K, Suvanto P, Davies J, Wartiovaara J, Wartiovaara K, et al (1997) Glial-cell-line-derived neurotrophic factor is required for bud initiation from ureteric epithelium. Development 124: 4077–4087. 9374404 10.1242/dev.124.20.4077

[6] Zhao H, Kegg H, Grady S, Truong HT, Robinson ML, et al (2004) Role of fibroblast growth factor receptors 1 and 2 in the ureteric bud. Dev Biol 276: 403–415. 15581874 10.1016/j.ydbio.2004.09.002 PMC4131686

[7] Michos O, Cebrian C, Hyink D, Grieshammer U, Williams L, et al (2010) Kidney development in the absence of Gdnf and Spry1 requires Fgf10. PLoS Genet 6: e1000809. 20084103 10.1371/journal.pgen.1000809 PMC2797609

[8] Song R, El-Dahr SS, Yosypiv IV (2011) Receptor tyrosine kinases in kidney development. J Signal Transduct 2011: 869281. 21637383 10.1155/2011/869281 PMC3100575

[9] Tang MJ, Cai Y, Tsai SJ, Wang YK, Dressler GR (2002) Ureteric bud outgrowth in response to RET activation is mediated by phosphatidylinositol 3-kinase. Dev Biol 243: 128–136. 11846482 10.1006/dbio.2001.0557

[10] Fisher CE, Michael L, Barnett MW, Davies JA (2001) Erk MAP kinase regulates branching morphogenesis in the developing mouse kidney. Development 128: 4329–4338. 11684667 10.1242/dev.128.21.4329

[11] Watanabe T, Costantini F (2004) Real-time analysis of ureteric bud branching morphogenesis in vitro. Dev Biol 271: 98–108. 15196953 10.1016/j.ydbio.2004.03.025

[12] Willecke R, Heuberger J, Grossmann K, Michos O, Schmidt-Ott K, et al (2011) The tyrosine phosphatase Shp2 acts downstream of GDNF/Ret in branching morphogenesis of the developing mouse kidney. Dev Biol 360: 310–317. 22015719 10.1016/j.ydbio.2011.09.029

[13] Kim D, Dressler GR (2007) PTEN modulates GDNF/RET mediated chemotaxis and branching morphogenesis in the developing kidney. Dev Biol 307: 290–299. 17540362 10.1016/j.ydbio.2007.04.051 PMC2129124

[14] Jain S, Encinas M, Johnson EM Jr, Milbrandt J (2006) Critical and distinct roles for key RET tyrosine docking sites in renal development. Genes Dev 20: 321–333. 16452504 10.1101/gad.1387206 PMC1361703

[15] Hoshi M, Batourina E, Mendelsohn C, Jain S (2012) Novel mechanisms of early upper and lower urinary tract patterning regulated by RetY1015 docking tyrosine in mice. Development 139: 2405–2415. 22627285 10.1242/dev.078667 PMC3367447

[16] de Graaff E, Srinivas S, Kilkenny C, D'Agati V, Mankoo BS, et al (2001) Differential activities of the RET tyrosine kinase receptor isoforms during mammalian embryogenesis. Genes Dev 15: 2433–2444. 11562352 10.1101/gad.205001 PMC312785

[17] Wong A, Bogni S, Kotka P, de Graaff E, D'Agati V, et al (2005) Phosphotyrosine 1062 is critical for the in vivo activity of the Ret9 receptor tyrosine kinase isoform. Mol Cell Biol 25: 9661–9673. 16227613 10.1128/MCB.25.21.9661-9673.2005 PMC1265823

[18] Michael L, Davies JA (2004) Pattern and regulation of cell proliferation during murine ureteric bud development. J Anat 204: 241–255. 15061751 10.1111/j.0021-8782.2004.00285.x PMC1571296

[19] Packard A, Georgas K, Michos O, Riccio P, Cebrian C, et al (2013) Luminal Mitosis Drives Epithelial Cell Dispersal within the Branching Ureteric Bud. Dev Cell 27: 319–330. 24183650 10.1016/j.devcel.2013.09.001 PMC3926506

[20] Chi X, Michos O, Shakya R, Riccio P, Enomoto H, et al (2009) Ret-dependent cell rearrangements in the Wolffian duct epithelium initiate ureteric bud morphogenesis. Dev Cell 17: 199–209. 19686681 10.1016/j.devcel.2009.07.013 PMC2762206

[21] Kuure S, Cebrian C, Machingo Q, Lu BC, Chi X, et al (2010) Actin depolymerizing factors cofilin1 and destrin are required for ureteric bud branching morphogenesis. PLoS Genet 6: e1001176. 21060807 10.1371/journal.pgen.1001176 PMC2965756

[22] Roskoski R Jr (2012) MEK1/2 dual-specificity protein kinases: structure and regulation. Biochem Biophys Res Commun 417: 5–10. 22177953 10.1016/j.bbrc.2011.11.145

[23] Roskoski R Jr (2012) ERK1/2 MAP kinases: structure, function, and regulation. Pharmacol Res 66: 105–143. 22569528 10.1016/j.phrs.2012.04.005

[24] Ishibe S, Joly D, Zhu X, Cantley LG (2003) Phosphorylation-dependent paxillin-ERK association mediates hepatocyte growth factor-stimulated epithelial morphogenesis. Mol Cell 12: 1275–1285. 14636584 10.1016/s1097-2765(03)00406-4

[25] Ishibe S, Joly D, Liu ZX, Cantley LG (2004) Paxillin serves as an ERK-regulated scaffold for coordinating FAK and Rac activation in epithelial morphogenesis. Mol Cell 16: 257–267. 15494312 10.1016/j.molcel.2004.10.006

[26] Peng X, Nelson ES, Maiers JL, DeMali KA (2011) New insights into vinculin function and regulation. Int Rev Cell Mol Biol 287: 191–231. 21414589 10.1016/B978-0-12-386043-9.00005-0 PMC4426885

[27] Dubrovskyi O, Tian X, Poroyko V, Yakubov B, Birukova AA, et al (2012) Identification of paxillin domains interacting with beta-catenin. FEBS Lett 586: 2294–2299. 22728435 10.1016/j.febslet.2012.06.016 PMC3407333

[28] le Duc Q, Shi Q, Blonk I, Sonnenberg A, Wang N, et al (2010) Vinculin potentiates E-cadherin mechanosensing and is recruited to actin-anchored sites within adherens junctions in a myosin II-dependent manner. J Cell Biol 189: 1107–1115. 20584916 10.1083/jcb.201001149 PMC2894457

[29] Peng X, Cuff LE, Lawton CD, DeMali KA (2010) Vinculin regulates cell-surface E-cadherin expression by binding to beta-catenin. J Cell Sci 123: 567–577. 20086044 10.1242/jcs.056432 PMC2818194

[30] Bissonauth V, Roy S, Gravel M, Guillemette S, Charron J (2006) Requirement for Map2k1 (Mek1) in extra-embryonic ectoderm during placentogenesis. Development 133: 3429–3440. 16887817 10.1242/dev.02526

[31] Belanger LF, Roy S, Tremblay M, Brott B, Steff AM, et al (2003) Mek2 is dispensable for mouse growth and development. Mol Cell Biol 23: 4778–4787. 12832465 10.1128/MCB.23.14.4778-4787.2003 PMC162209

[32] Scholl FA, Dumesic PA, Barragan DI, Harada K, Bissonauth V, et al (2007) Mek1/2 MAPK kinases are essential for Mammalian development, homeostasis, and Raf-induced hyperplasia. Dev Cell 12: 615–629. 17419998 10.1016/j.devcel.2007.03.009

[33] Lu BC, Cebrian C, Chi X, Kuure S, Kuo R, et al (2009) Etv4 and Etv5 are required downstream of GDNF and Ret for kidney branching morphogenesis. Nat Genet 41: 1295–1302. 19898483 10.1038/ng.476 PMC2787691

[34] Ola R, Jakobson M, Kvist J, Perala N, Kuure S, et al (2011) The GDNF Target Vsnl1 Marks the Ureteric Tip. J Am Soc Nephrol 22: 274–284. 21289216 10.1681/ASN.2010030316 PMC3029900

[35] Cabrita MA, Christofori G (2008) Sprouty proteins, masterminds of receptor tyrosine kinase signaling. Angiogenesis 11: 53–62. 18219583 10.1007/s10456-008-9089-1

[36] Chambard JC, Lefloch R, Pouyssegur J, Lenormand P (2007) ERK implication in cell cycle regulation. Biochim Biophys Acta 1773: 1299–1310. 17188374 10.1016/j.bbamcr.2006.11.010

[37] Shinohara M, Mikhailov AV, Aguirre-Ghiso JA, Rieder CL (2006) Extracellular signal-regulated kinase 1/2 activity is not required in mammalian cells during late G2 for timely entry into or exit from mitosis. Mol Biol Cell 17: 5227–5240. 17035635 10.1091/mbc.E06-04-0284 PMC1679686

[38] Guadagno TM, Ferrell JE Jr (1998) Requirement for MAPK activation for normal mitotic progression in Xenopus egg extracts. Science 282: 1312–1315. 9812894 10.1126/science.282.5392.1312

[39] Horne MM, Guadagno TM (2003) A requirement for MAP kinase in the assembly and maintenance of the mitotic spindle. J Cell Biol 161: 1021–1028. 12821640 10.1083/jcb.200304144 PMC2172988

[40] Lavoie JN, L'Allemain G, Brunet A, Muller R, Pouyssegur J (1996) Cyclin D1 expression is regulated positively by the p42/p44MAPK and negatively by the p38/HOGMAPK pathway. J Biol Chem 271: 20608–20616. 8702807 10.1074/jbc.271.34.20608

[41] Weber JD, Raben DM, Phillips PJ, Baldassare JJ (1997) Sustained activation of extracellular-signal-regulated kinase 1 (ERK1) is required for the continued expression of cyclin D1 in G1 phase. Biochem J 326 (Pt 1): 61–68. 9337851 10.1042/bj3260061 PMC1218637

[42] Barasch J, Pressler L, Connor J, Malik A (1996) A ureteric bud cell line induces nephrogenesis in two steps by two distinct signals. Am J Physiol 271: F50–F61. 8760243 10.1152/ajprenal.1996.271.1.F50

[43] Kuure S (2012) Analysis of migration in primary ureteric bud epithelial cells. Methods Mol Biol 886: 147–155. 22639258 10.1007/978-1-61779-851-1_13

[44] Cavey M, Rauzi M, Lenne PF, Lecuit T (2008) A two-tiered mechanism for stabilization and immobilization of E-cadherin. Nature 453: 751–756. 18480755 10.1038/nature06953

[45] Paredes J, Figueiredo J, Albergaria A, Oliveira P, Carvalho J, et al (2012) Epithelial E- and P-cadherins: role and clinical significance in cancer. Biochim Biophys Acta 1826: 297–311. 22613680 10.1016/j.bbcan.2012.05.002

[46] Keil R, Schulz J, Hatzfeld M (2013) p0071/PKP4, a multifunctional protein coordinating cell adhesion with cytoskeletal organization. Biol Chem 394: 1005–1017. 23640939 10.1515/hsz-2013-0114

[47] Li J, Ananthapanyasut W, Yu AS (2011) Claudins in renal physiology and disease. Pediatr Nephrol 26: 2133–2142. 21365189 10.1007/s00467-011-1824-y PMC3203223

[48] Nadeau V, Guillemette S, Belanger LF, Jacob O, Roy S, et al (2009) Map2k1 and Map2k2 genes contribute to the normal development of syncytiotrophoblasts during placentation. Development 136: 1363–1374. 19304888 10.1242/dev.031872

[49] Lefloch R, Pouyssegur J, Lenormand P (2008) Single and combined silencing of ERK1 and ERK2 reveals their positive contribution to growth signaling depending on their expression levels. Mol Cell Biol 28: 511–527. 17967895 10.1128/MCB.00800-07 PMC2223286

[50] Costantini F (2010) GDNF/Ret signaling and renal branching morphogenesis: From mesenchymal signals to epithelial cell behaviors. Organogenesis 6: 252–262. 21220964 10.4161/org.6.4.12680 PMC3055651

[51] Lewellis SW, Knaut H (2012) Attractive guidance: how the chemokine SDF1/CXCL12 guides different cells to different locations. Semin Cell Dev Biol 23: 333–340. 22414535 10.1016/j.semcdb.2012.03.009 PMC3345092

[52] Meloche S, Pouyssegur J (2007) The ERK1/2 mitogen-activated protein kinase pathway as a master regulator of the G1- to S-phase transition. Oncogene 26: 3227–3239. 17496918 10.1038/sj.onc.1210414

[53] de Beco S, Amblard F, Coscoy S (2012) New insights into the regulation of E-cadherin distribution by endocytosis. Int Rev Cell Mol Biol 295: 63–108. 22449487 10.1016/B978-0-12-394306-4.00008-3

[54] Fujita Y, Krause G, Scheffner M, Zechner D, Leddy HE, et al (2002) Hakai, a c-Cbl-like protein, ubiquitinates and induces endocytosis of the E-cadherin complex. Nat Cell Biol 4: 222–231. 11836526 10.1038/ncb758

[55] Quadri SK (2012) Cross talk between focal adhesion kinase and cadherins: role in regulating endothelial barrier function. Microvasc Res 83: 3–11. 21864544 10.1016/j.mvr.2011.08.001 PMC3427140

[56] Serrels A, Canel M, Brunton VG, Frame MC (2011) Src/FAK-mediated regulation of E-cadherin as a mechanism for controlling collective cell movement: insights from in vivo imaging. Cell Adh Migr 5: 360–365. 21836391 10.4161/cam.5.4.17290 PMC3210304

[57] Thomas WA, Boscher C, Chu YS, Cuvelier D, Martinez-Rico C, et al (2013) alpha-Catenin and vinculin cooperate to promote high E-cadherin-based adhesion strength. J Biol Chem 288: 4957–4969. 23266828 10.1074/jbc.M112.403774 PMC3576099

[58] Kuure S, Sainio K, Vuolteenaho R, Ilves M, Wartiovaara K, et al (2005) Crosstalk between Jagged1 and GDNF/Ret/GFRalpha1 signalling regulates ureteric budding and branching. Mech Dev 122: 765–780. 15905075 10.1016/j.mod.2005.03.006

[59] Wilkinson DG (1992) Whole mount in situ hybridization of vertebrate embryos. In: Wilkinson DG, editor. In situ hybridization: a practical approach. Oxford: IRL Press. pp. 75–83.

[60] Runeberg-Roos P, Virtanen H, Saarma M (2007) RET(MEN 2B) is active in the endoplasmic reticulum before reaching the cell surface. Oncogene 26: 7909–7915. 17599050 10.1038/sj.onc.1210591

