# Supplementary figures and images for: Mitogen-Activated Protein Kinase (MAPK) Pathway Regulates Branching by Remodeling Epithelial Cell Adhesion
Source: PLoS Genet. 2014 Mar 6;10(3):e1004193. doi: 10.1371/journal.pgen.1004193 (PMC3945187; doi:10.1371/journal.pgen.1004193)

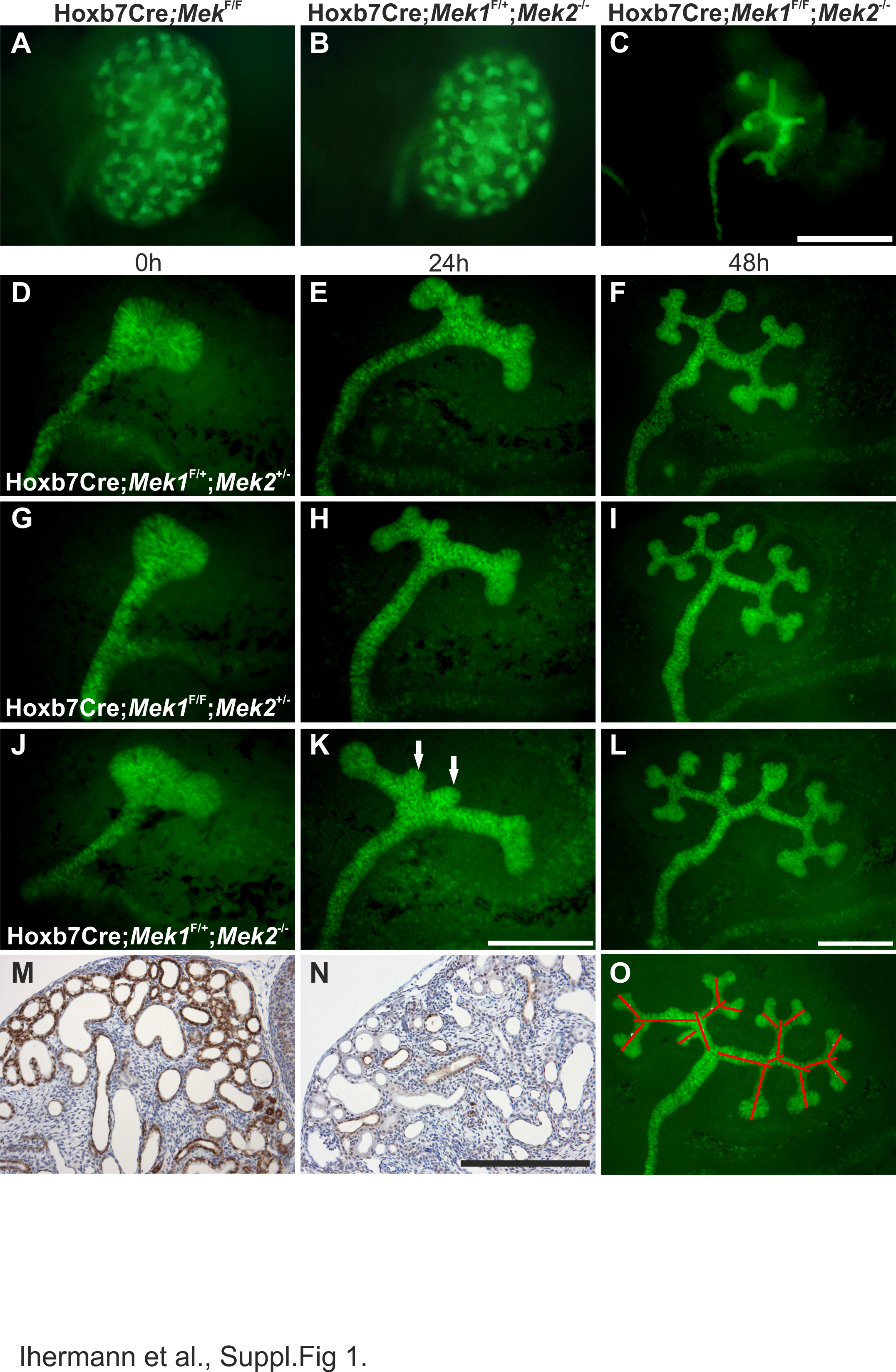

Supplement: Figure S1 — Ureteric bud branching in the absence of, or with reduced, MAPK pathway activity. (A) Deletion of Mek1 alone specifically in the UB epithelium has no effect on renal development as seen by the normal morphology and branching pattern in E13.5 Hoxb7CreGFP;Mek1F/F kidney, where the UB is visualized by the GFP. (B) Deletion of three out of four Mek1 and -2 alleles (in any combination) is enough to support normal growth and branching of the UB as shown by GFP in E13.5 Hoxb7CreGFP;Mek1F/+;Mek2-/- kidney. (C) Severely reduced UB branching morphogenesis in the absence of MAPK activity is illustrated by GFP-tag in UB of dko kidney. (D–L) In vitro organ culture of (D–F) control kidneys and (G–L) those lacking three out of four Mek1 and -2 alleles show no differences in their capacity to form new branches during the 48 h observation period. (M) Na/K ATPase and (N) Tamm-Horsefall staining in newborn dko kidney. (O) Illustration of stalk length measurements from E11.5 wild-type kidneys cultured for 48 h where the length of every UB stalk is measured from one branch point to the next. Scale bars 500 µm. (TIF) [file pgen.1004193.s001.tif]

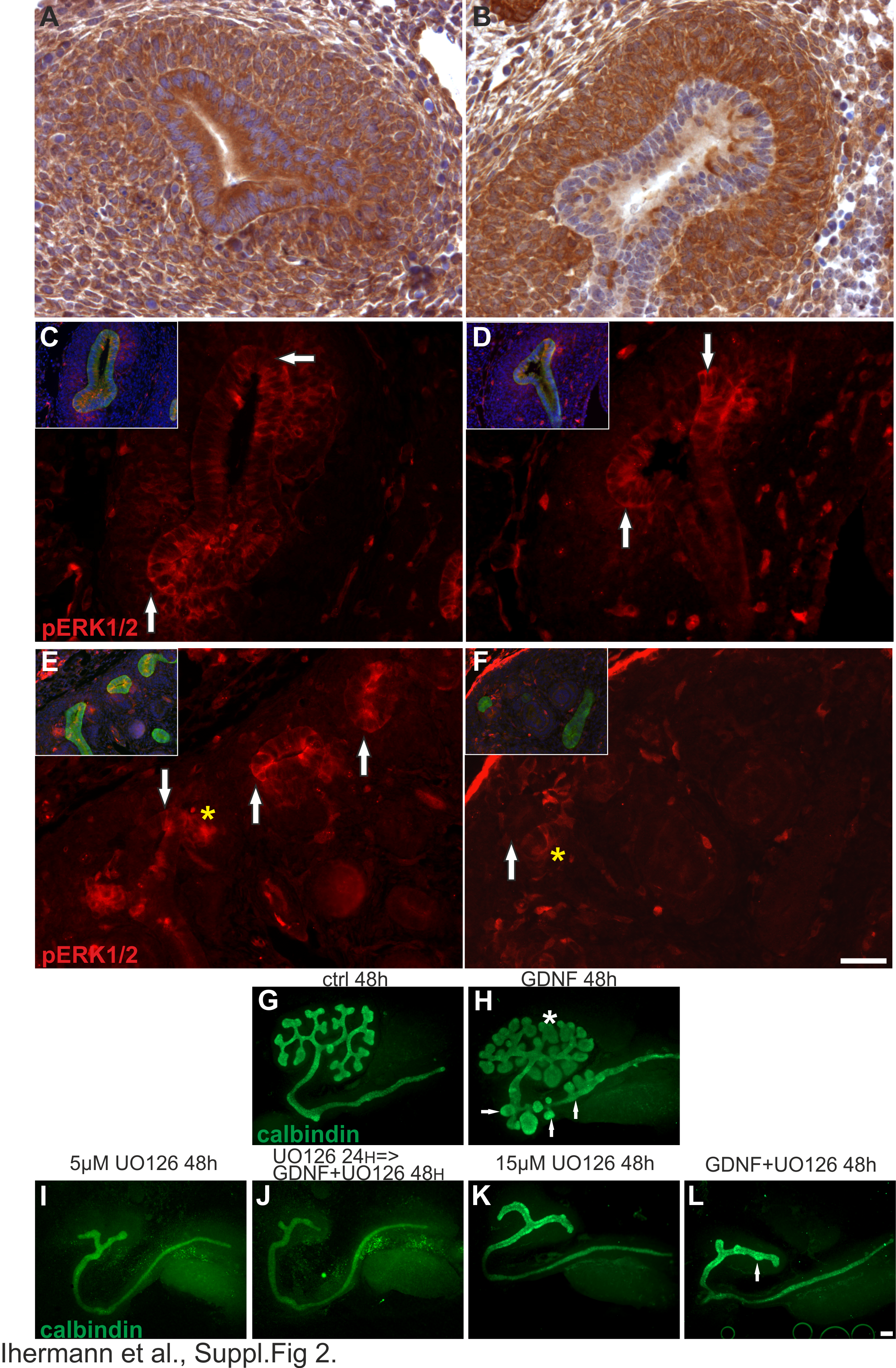

Supplement: Figure S2 — MAPK pathway activity and formation of primary ureteric bud. (A) MEK1 is ubiquitously distributed in E11.5 control kidney, while it is missing from most UB epithelial cells in (B) dko UB. (C) Prominent MAPK activity detected by pERK1/2 antibody staining (red) is observed in early ureteric bud (arrows) formed in E11.5 control embryo (n = 4). (D) MAPK activity (red) is maintained in UB epithelium (arrows) of dko kidney at E11.5 (n = 5). (E) The pattern of pERK1/2 staining in E13.5 control kidney demonstrates MAPK pathway activity in UB tips (arrows) and freshly induced nephron primordia (asterisk). (F) The double knockout UB tip (arrow) is depleted of pERK1/2 staining (red) although it is maintained in newly induced nephron primordia (asterisk). Insets in (C–F) show the corresponding sections labeled with UB epithelial marker calbindin (green). (G) E11.5 control kidney cultured for 48 h and stained with calbindin (green) to visualize UB epithelium shows renal-type branching pattern, which is sustained (H) in the presence of exogenous GDNF (100 ng/ml). Extra GDNF causes typical dilatation of UB tips (asterisk) and ectopic bud formation from the nephric duct (arrows) mimicking the normal bud formation in the earliest stage of kidney development. (I) Chemical inhibition of MAPK pathway with low (5 µM) concentration of UO126 in cultured E11.5 kidney disturbs the normal branching of the UB. (J) Pretreatment of E11.5 kidneys with 5 µM UO126 followed by addition of GDNF (100 ng/ml) in the presence of MEK-inhibitor (5 µM) inhibits normal branching and typical response to extra GDNF. (K) Inhibition of MEK1/2 with 15 µM UO126 blocked formation of new UB branches. (L) Simultaneous inhibition of MAPK pathway and GDNF stimulus blocks the normal effects of exogenous GDNF. Scale bars: A–D 100 µm, E–J 1 mm. (TIF) [file pgen.1004193.s002.tif]

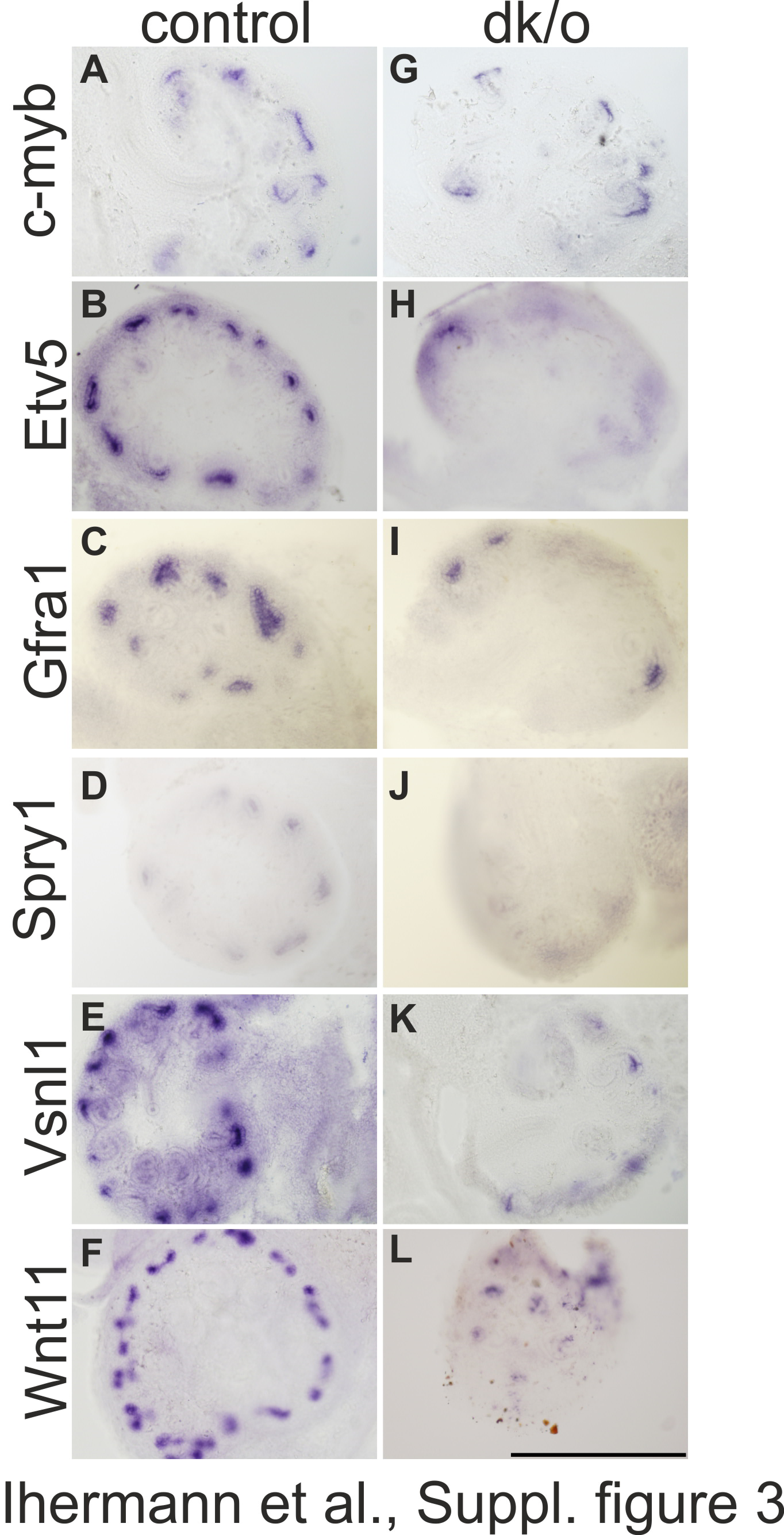

Supplement: Figure S3 — Expression of GDNF target genes in UB epithelium. E13.5 vibratome sections of (A–F) control and (G–L) dko kidneys were hybridized with (A, G) c-myb, (B, H) Etv5, (C, I) Gfrα1, (D, J) Spry1, (E, K) Vsnl1 and (F, L) Wnt11. Scale bar: 500 µm. (TIF) [file pgen.1004193.s003.tif]

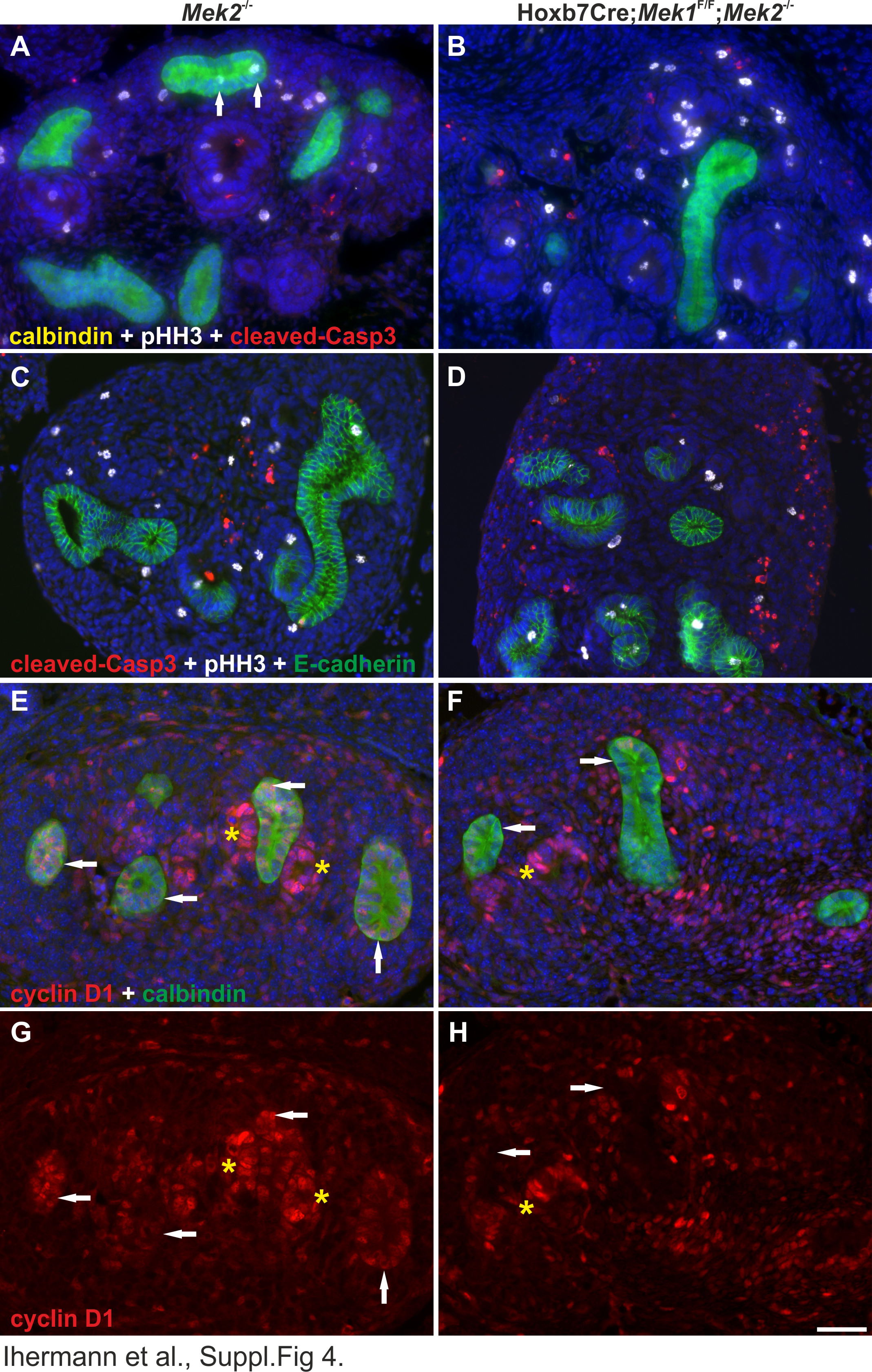

Supplement: Figure S4 — Apoptosis and proliferation in the absence of MAPK pathway activity. (A) E14.5 control and (B) dko kidneys stained with pHH3 (white), cleaved-Casp3 (red) and UB-marker calbindin (green). UB epithelial cells in mitosis are indicated by arrows. (C) E12.5 control and (D) dko kidneys stained with markers of cell death (cleaved-Casp3, red), proliferation (pHH3, white) and epithelium (E-cadherin, green) show comparable patterns of apoptotic and mitotic nuclei. (E) ERK target cyclin D1 (red), which is required for G1/S transition during the cell cycle progress, is found in differentiating nephron primordia (asterisk) and in most of the UB epithelial cells (green) of E12.5 control kidney. (F) The vast majority of UB cells (green) lack cyclin D1 in dko kidneys while its pattern in nephron primordia (asterisk) remains normal. (G–H) Images showing only cyclin D1 staining seen in E and F, respectively. Arrows indicate the UB epithelium. Scale bar: 50 µm. (TIF) [file pgen.1004193.s004.tif]

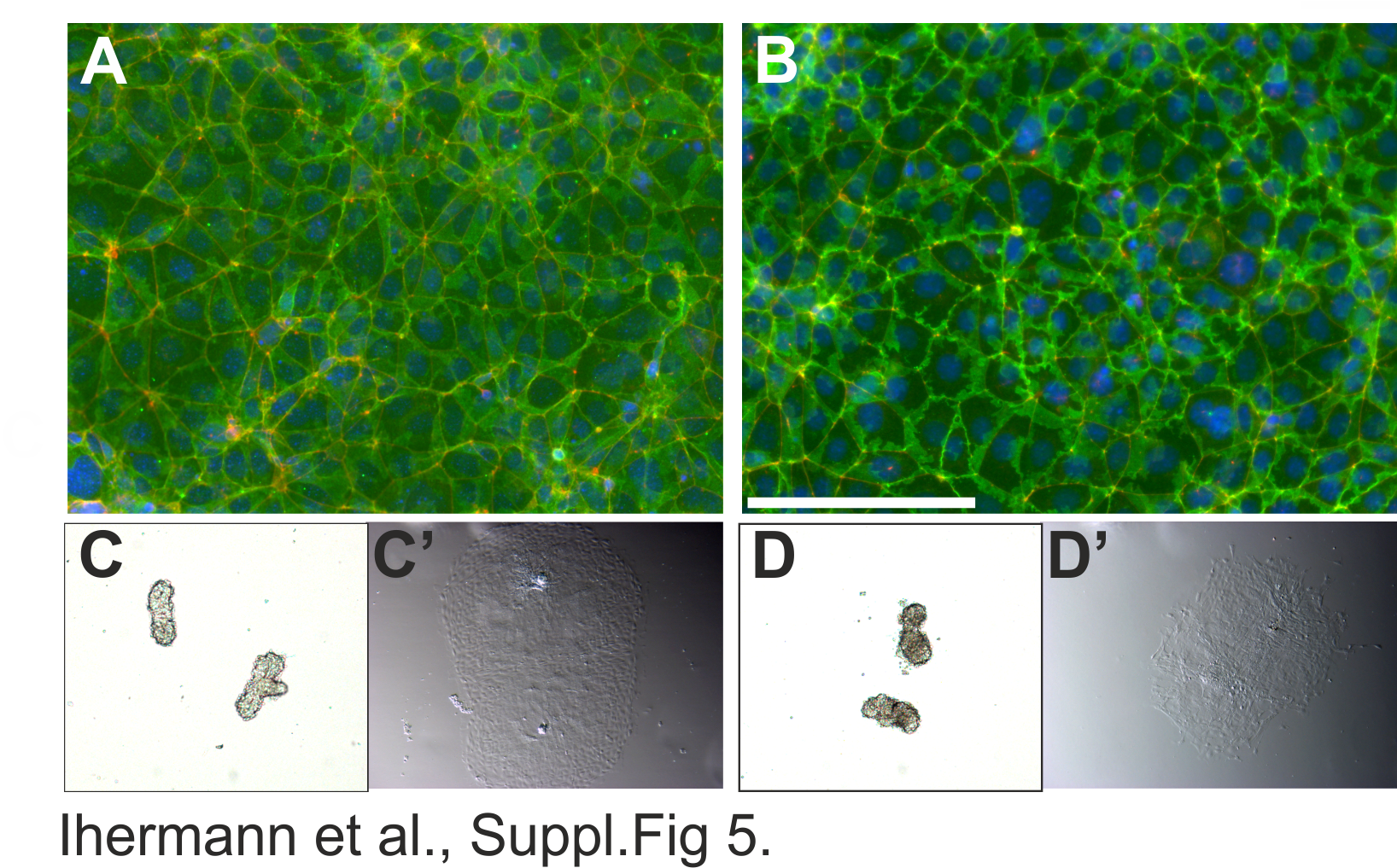

Supplement: Figure S5 — Low magnification images of those shown at higher magnification in Figure 7C–F. (A) control and (B) UO126-treated UB cells stained for E-cadherin (green) and F-actin visualized by Alexa568-conjugated phalloidin (red) show more E-cadherin on cell membranes. (C–D) Ureteric buds isolated from E11.5 (C) control and (D) dko kidneys cultured for 48 h (C′ and D′, respectively) to set-up monolayer cultures. Scale bars: 50 µm. (TIF) [file pgen.1004193.s005.tif]

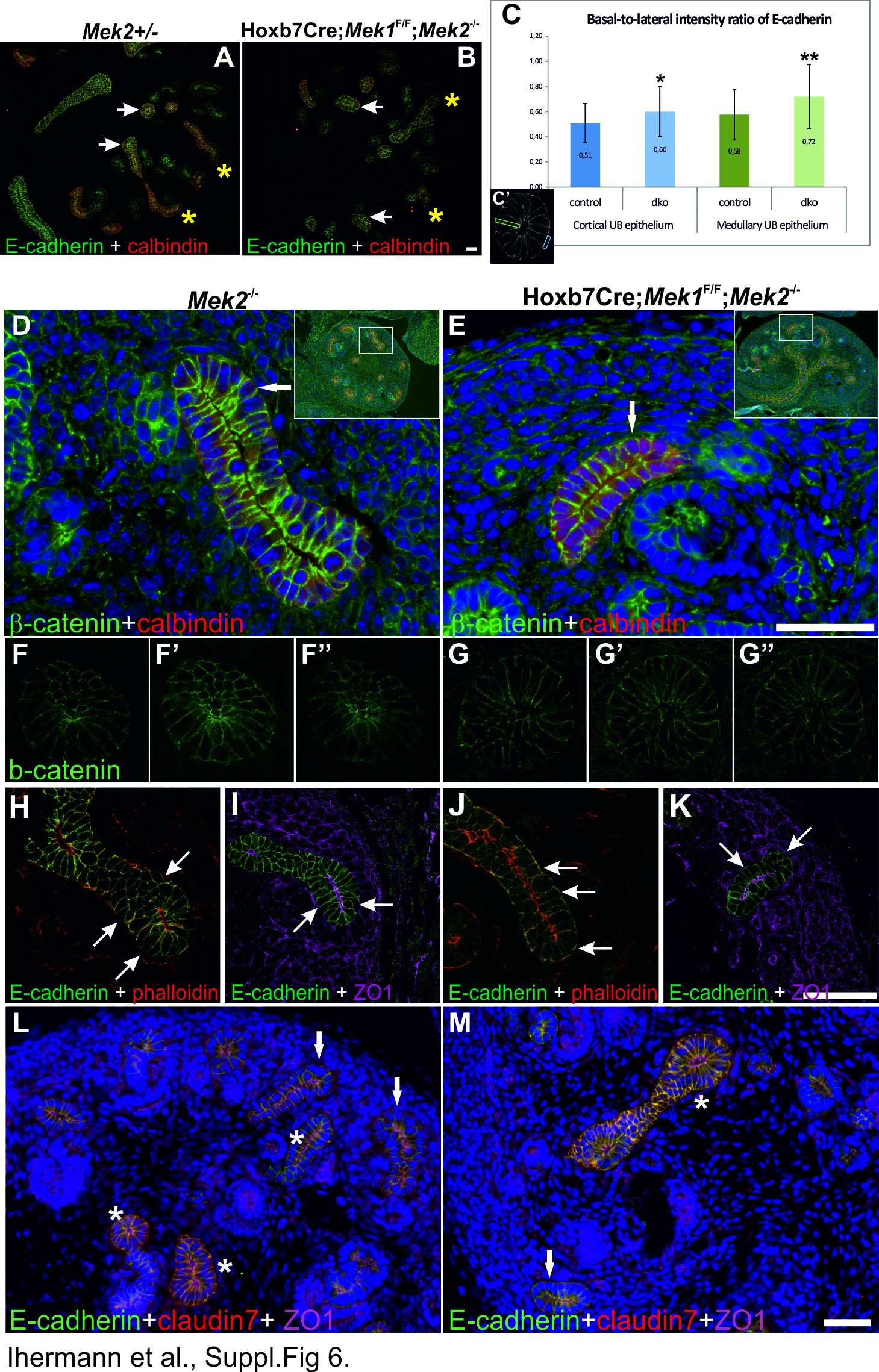

Supplement: Figure S6 — Adherens junction changes appear specific to E-cadherin. Merged confocal image of E13.5 (A) control and (B) dko kidneys stained with calbindin (red) to illustrate ureteric epithelium, and E-cadherin (green) to show the cell-cell contacts. Arrows point to the medullary, more mature UB with stronger E-cad intensity, asterisk point UB tips. (C) Average basal-to-lateral ratios of E-cadherin intensities in control (blue and green) and dko (light blue and light green) UB epithelial cells. In the inset C′, the corresponding cell membranes are indicated in blue and green, respectively. The intensities in cortical and medullary UB epithelium are stronger in the absence of MAPK activtity (*p<0.05 and **p<0.01, respectively, 2-tailed T-test, n = 70 cells in seven ctrl UBs, and 97 cells in 11 dko UBs). (D) β-catenin (green) localization in E13.5 control and (E) dko UBs (calbindin, red). Insets in D and E show the cross section of entire kidney at E13.5, and boxed area is enlarged in the actual image. Confocal stack images of β-catenin localization in cell membranes of (F–F″) control and (G–G″) dko UB. (H) Phalloidin (red) is distributed to apical and basal membranes of UB epithelium and shows very little if any co-localization with E-cadherin (green) in basal membranes of E13.5 control UB epithelium. (I) Double staining of E-cadherin (green) and apical cell polarity marker ZO1 (purple) in E13.5 UB of control kidney. (J) Several sites of co-localization (yellow) and domination of green signal (arrows) demonstrate E-cadherin localization on basal membrane of UB deficient for MAPK pathway. (K) UB epithelium of E13.5 dko kidneys show no changes in apical polarization (ZO1, purple), while demonstrating the mis-localization of E-cadherin to basal membranes in mutant epithelium (arrows). (L) Another junction protein, Claudin-7 (red), is co-expressed with E-cadherin (green) in medullary regions (asterisks) of control UB and (M) MAPK pathway deficient UB. ZO1 is shown in purple, arrows [file pgen.1004193.s006.tif]

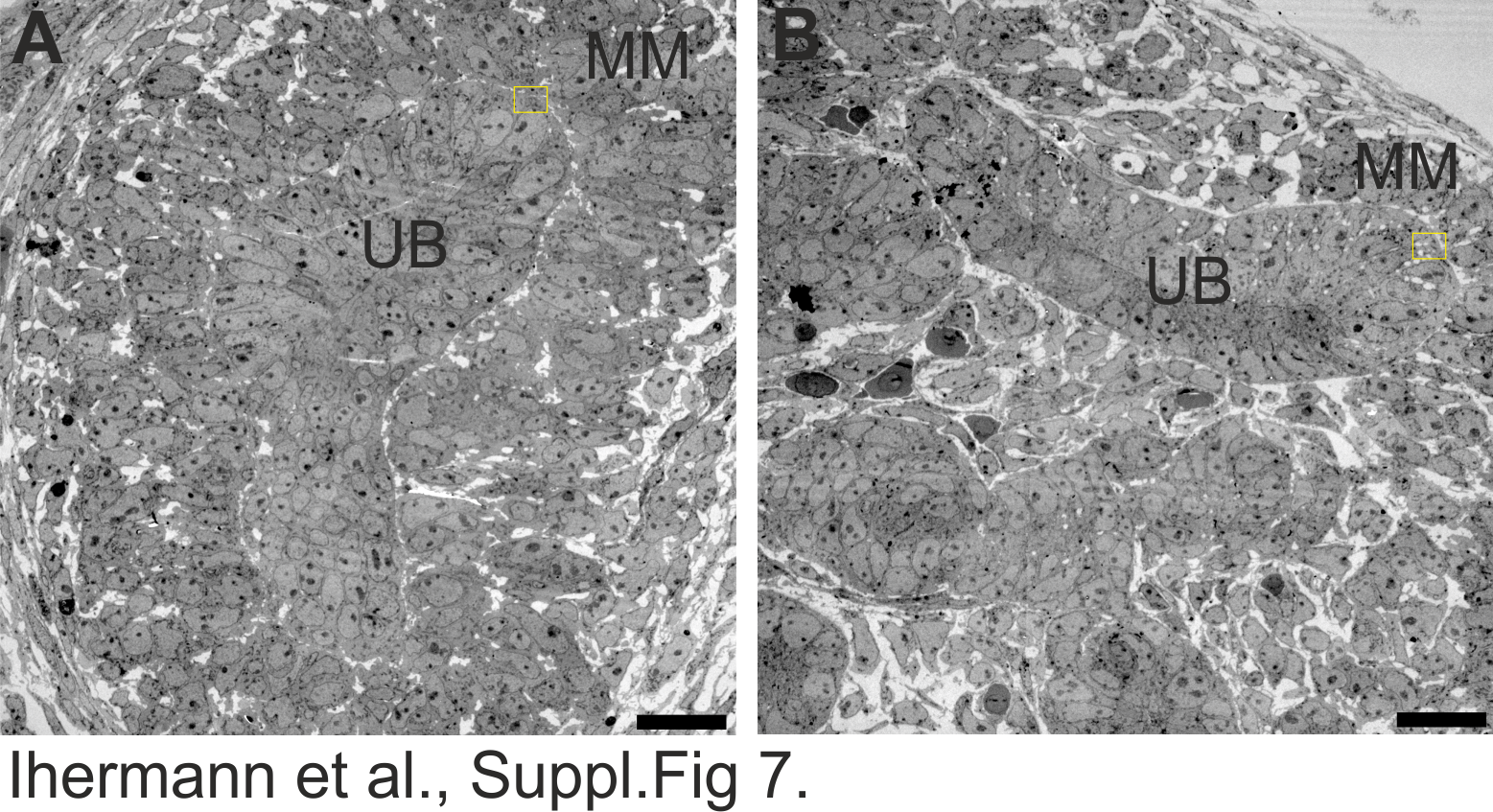

Supplement: Figure S7 — Low magnification electron microscopy (EM) image of E12.5 (A) control and (B) dko kidneys, where yellow rectangles indicate the regions of UBs magnified in Figure 8E–F, respectively. Scale bars: 50 µm. (TIF) [file pgen.1004193.s007.tif]
